# The role of CenKR in the coordination of *Rhodobacter sphaeroides* cell elongation and division

**DOI:** 10.1128/mbio.00631-23

**Published:** 2023-06-07

**Authors:** Bryan D. Lakey, François Alberge, Daniel Parrell, Elizabeth R. Wright, Daniel R. Noguera, Timothy J. Donohue

**Affiliations:** 1 Wisconsin Energy Institute, Great Lakes Bioenergy Research Center, University of Wisconsin-Madison, Madison, Wisconsin, USA; 2 Laboratory of Genetics, University of Wisconsin-Madison, Madison, Wisconsin, USA; 3 Department of Biochemistry, University of Wisconsin-Madison, Madison, Wisconsin, USA; 4 Cryo-Electron Microscopy Research Center,Department of Biochemistry, University of Wisconsin-Madison, Madison, Wisconsin, USA; 5 Midwest Center for Cryo-Electron Tomography, Department of Biochemistry, University of Wisconsin-Madison, Madison, Wisconsin, USA; 6 Department of Civil and Environmental Engineering, University of Wisconsin-Madison, Madison, Wisconsin, USA; 7 Department of Bacteriology, University of Wisconsin-Madison, Madison, Wisconsin, USA; University of Washington School of Medicine, Seattle, Washington, USA

**Keywords:** *Rhodobacter*, cell division, gram-negative cell envelope, tol-pal, two-component system, alphaproteobacteria, cryo-electron microscopy, cryo-electron tomography

## Abstract

Cell elongation and division are essential aspects of the bacterial life cycle that must be coordinated for viability and replication. The impact of misregulation of these processes is not well understood as these systems are often not amenable to traditional genetic manipulation. Recently, we reported on the CenKR two-component system (TCS) in the Gram-negative bacterium *Rhodobacter sphaeroides* that is genetically tractable, widely conserved in α-proteobacteria, and directly regulates the expression of components crucial for cell elongation and division, including genes encoding subunit of the Tol-Pal complex. In this work, we show that overexpression of *cenK* results in cell filamentation and chaining. Using cryo-electron microscopy (cryo-EM) and cryo-electron tomography (cryo-ET), we generated high-resolution two-dimensional (2D) images and three-dimensional (3D) volumes of the cell envelope and division septum of wild-type cells and a *cenK* overexpression strain finding that these morphological changes stem from defects in outer membrane (OM) and peptidoglycan (PG) constriction. By monitoring the localization of Pal, PG biosynthesis, and the bacterial cytoskeletal proteins MreB and FtsZ, we developed a model for how increased CenKR activity leads to changes in cell elongation and division. This model predicts that increased CenKR activity decreases the mobility of Pal, delaying OM constriction, and ultimately disrupting the midcell positioning of MreB and FtsZ and interfering with the spatial regulation of PG synthesis and remodeling.

**IMPORTANCE:**

By coordinating cell elongation and division, bacteria maintain their shape, support critical envelope functions, and orchestrate division. Regulatory and assembly systems have been implicated in these processes in some well-studied Gram-negative bacteria. However, we lack information on these processes and their conservation across the bacterial phylogeny. In *R. sphaeroides* and other α-proteobacteria, CenKR is an essential two-component system (TCS) that regulates the expression of genes known or predicted to function in cell envelope biosynthesis, elongation, and/or division. Here, we leverage unique features of CenKR to understand how increasing its activity impacts cell elongation/division and use antibiotics to identify how modulating the activity of this TCS leads to changes in cell morphology. Our results provide new insight into how CenKR activity controls the structure and function of the bacterial envelope, the localization of cell elongation and division machinery, and cellular processes in organisms with importance in health, host-microbe interactions, and biotechnology.

## INTRODUCTION

Gram-negative cells contain a diderm membrane system consisting of an inner membrane (IM) phospholipid bilayer and an outer membrane (OM) that is composed of an inner leaflet of phospholipids and an outer leaflet of lipopolysaccharide (LPS) molecules (1). The two membranes are separated by a periplasmic space containing a thin layer of peptidoglycan (PG) that is closely associated with the OM via both covalent and non-covalent connections maintained by OM proteins and lipoproteins (1). The synthesis of cell envelope materials, specifically PG, is critical for cell shape, elongation, and division. While much is known about the function, assembly, and spatiotemporal movement of macromolecular complexes that are essential for cell elongation [elongasome (2)] and division [divisome (3)] in several well-studied bacteria, little is known about the coordination of these processes. We are interested in the coordination of these processes in α-proteobacteria, a bacterial group that displays a wide variety of cell elongation or division patterns (4), lacks homologous post-translational regulators such as OM lipoproteins that have been described in *Escherichia coli* and other Gram-negative bacteria (5 - 7), and have properties of biomedical, environmental, agricultural, and industrial importance.

In addition to the considerable understanding of the molecular mechanisms of PG biosynthesis that underlie cell elongation and division in the γ-proteobacterium *E. coli*, the coordination of OM constriction during division has also been recently described (8 - 10). In this bacterium, the IM TolQRA complex is localized to the midcell during septum formation by interactions with subunits of the divisome (8, 9). At the time of cell division, TolQRA interacts with the TolB-Pal subcomplex to release the lipoprotein Pal, allowing it to populate the division septum and bind PG (9). These events are followed by constriction of the OM at the site of septum formation through non-covalent interactions of Pal with newly synthesized septal PG during the late stages of cell division (8, 9). The dynamic localization of *E. coli* TolQRA also regulates the activity of lateral PG synthesis complexes (PBP1B-LpoB-CpoB) during cell elongation (6) as well as the hydrolysis of cell septum PG strands ensuring separation of the daughter cells after cell division (11). However, α-proteobacteria lack homologs to several of the proteins implicated in these important “secondary” functions. In addition, unlike the situation in *E. coli* which divide by binary fission (12), α-proteobacteria display a wide-variety of budding morphologies, midcell constriction, as well as asymmetric or polar growth, which likely requires fundamentally different cell elongation and division machinery (4). This diversity of cell properties may also explain why the genes encoding several subunits of the Tol-Pal complex (*tolQ*, *tolA*, *tolR*, *tolB*, and *pal*) are known or predicted to be essential in *R. sphaeroides* (13) and other α-proteobacteria (14, 15), unlike the case in *E. coli* and several other bacteria.

Often, important insights into cell envelope processes, elongation, and division have come from analyzing the role of TCSs. However, TCSs that have been shown to indirectly impact the expression of cell envelope genes in *E. coli* (16, 17) are not typically conserved outside of γ-proteobacteria. Moreover, the TCSs that directly regulate the expression of cell envelope processes have been described in few microbes and are generally not well conserved (18 - 21). The essential nature of TCSs that regulate cell cycle processes in some α-proteobacteria has made it difficult to dissect specific roles in cell envelope biosynthesis and division due to pleiotropic effects (22 - 24). Consequently, less is known about envelope biosynthesis and the molecular impacts of these TCSs on cell elongation and division in α-proteobacteria.

Recently, we reported on the cell envelope histidine kinase and response regulator (CenKR) TCS in *R. sphaeroides* (25). We showed that the CenKR TCS is conserved in many α-proteobacteria and that CenR directly regulates transcription of genes encoding subunits of the TolQRAB complex as well as homologs of other proteins that are known or predicted to function in cell envelope biogenesis and division (25). In this work, we take advantage of the features of the *R. sphaeroides* CenKR TCS to study the dynamics of cell elongation and division in this α-proteobacterium. We show that ectopic expression of *cenK* increased activity of the CenKR TCS, leading to filamentation and cell chaining, as well as changes in the morphology of the periplasm and the OM. We used fluorescence microscopy to track changes in the temporal and spatial dynamics of PG biosynthesis, movement, and sequestration of Pal, and the localization of elongasome (MreB) and divisome (FtsZ) organizing filaments between wild-type cells and those with increased CenKR activity. Our results have provided important new insight into the processes of cell elongation and division in *R. sphaeroides* and allow us to propose how increased CenKR activity leads to the observed morphological changes. This work also lays a foundation for future studies to obtain a deeper understanding of cell elongation and division in this and other α-proteobacteria.

## MATERIALS AND METHODS

### Bacterial strains and growth conditions

*R. sphaeroides* strains (Table S1) were grown in Sistrom’s (SIS) minimal medium (26). Unless specified, cultures of 10 mL were grown in 125 mL flasks with shaking at 200 rpm, at 30°C for ~18 h until cells reached an OD600 of ~0.5 (mid-exponential growth). *E. coli* strains (Table S1) were grown at 37°C in Luria-Bertani medium. As needed, media was supplemented with 50 µg/mL kanamycin and/or 10 µM IPTG. For growth experiments, cell density was measured using a Klett-Summerson colorimeter equipped with a number 66 filter.

For treatment with previously determined (27) sub-MIC A22 (10 µg/mL, Cayman Chemical) or amdinocillin (mecillinam 0.5 µg/mL, Fisher Scientific), a single colony of *R. sphaeroides* was grown in SIS medium overnight. Cell cultures were then diluted to an OD600 ~0.05 with the addition of antibiotic and IPTG (10 µM) as appropriate. Cells were grown for 6 h (~2 doubling times) before imaging. For FM 4-64 stain (Setareh Biotech), a final concentration of 1 µg/mL was added to a small portion of culture before imaging (see below).

### Strain construction

All strains, primers, and plasmids used in this study are listed in Table S1. Construction of pIND5-cenR or pIND5-cenK was performed by PCR amplification of *cenR* or *cenK* from *R. sphaeroides* genomic DNA using Herculase II Fusion DNA Polymerase (Agilent). PCR products were assembled into PCR linearized pIND5 vector by Gibson Assembly (New England BioLabs) and transformed into DH5α cells (New England BioLabs). Transformants were screened by colony PCR and sequenced to confirm there were no mutations in coding regions. Plasmids were mobilized into *R. sphaeroides* via conjugal mating with *E. coli* S17-1 (28). Colony PCR of Kan^R^ *R. sphaeroides* colonies was used to confirm successful mobilization of pIND5-*cenR* or pIND5-*cenK*. Translational fusions to fluorescent reporters were constructed by allelic exchange using the suicide vector pk18*mobsacB* (29) as described previously (25). Gene insertions were confirmed by colony PCR of chromosomal loci and sequencing of genomic DNA with gene-specific primers (Table S1).

### Serial dilution spot titer assays

A single colony of *R. sphaeroides* was inoculated into 5 mL SIS medium with antibiotic as needed and was grown with shaking at 30°C overnight. Once cultures reached an OD600 of 0.4–0.6, they were diluted to an OD600 of 0.3 in 1 mL SIS. In a 96-well plate, 10 µL of dilute culture was serially diluted into 90 µL SIS. Next, 5 µL of each dilution series was spotted onto SIS agar plates containing ampicillin (2 µg/mL) or amdinocillin (1 µg/mL), and when appropriate IPTG (10 µM). Plates were allowed to dry and were incubated at 30°C until growth was observed (~2 days). Spot titer SIS plates were made fresh the day they were used, and antibiotics were added directly to melted SIS agar. Plates were dried at room temperature for 3 h before use. Spot titers were performed in triplicate.

### Fluorescence microscopy

All strains were grown as described above to an OD600 ~0.5. Cells (1 µL of culture) were immobilized on a coverslip under a 1.5% agarose gel pad. Images were taken using an EVOS FL microscopy with 100X oil immersion PLAN Apochromat objective (numerical aperture, 1.40). The EVOS light cube Texas Red (for mCherry and RADA signal) and DAPI (for HADA signal) were used to capture fluorescence signals. For analysis and cell segmentation, brightfield pictures were treated in FIJI (30) with the following settings: bandpass (large filter, 40 pixels; small filter, 2 pixels), background subtraction (rolling ball radius = 20 pixels), and contrast enhancing with normalization (0.1%). Fluorescence pictures were only treated with background subtraction (radius = 50 pixels). Segmentation was performed using the plugin MicrobeJ (31), and cell segmentation errors were manually removed, cell shape and fluorescence parameters were extracted. Demographs and maxima heatmaps were made with MicrobeJ. To monitor fluorescence levels in each strain, images were taken at the same exposure and light intensity with no background subtraction applied. The mean fluorescence per cell was calculated using MicrobeJ. Cell constriction’s relative positions were detected in MicrobeJ and manually verified for errors using FM4-64 staining. Figures and statistical analysis were performed in Rstudio using ggplot2 package (32).

### Cryo-electron microscopy and cryo-electron tomography

A 5 µL sample of each culture was sequentially deposited three times onto glow discharged 200 mesh *R2*/1 copper Quantifoil grids for 1 min. The first two 5 µL applications were manually blotted away after 1 min using Whatman number 1 filter paper before the next 5 µL application. The third 5 µL sample was applied along with 4 µL of 10 nm BSA Gold tracers (Electron Microscopy Sciences). After a 1 min incubation, the sample was blotted and plunged frozen in liquid ethane using a Vitrobot Mark IV (ThermoFisher Scientific). Cryo-EM and cryo-ET data were collected using a Titan Krios G3 TEM (ThermoFisher Scientific) operated at 300 kV, equipped with a Gatan Bioquantum GIF-K3 camera (Gatan, Inc.) in EFTEM mode using a 20 eV slit and a magnification of 19,500× (4.603 Å/pixel) or a Titan Krios G4i TEM (ThermoFisher Scientific) operated at 300 kV, equipped with a SelectrisX energy filter and a Falcon 4i camera (ThermoFisher Scientific) in EFTEM mode using a 10 eV slit and a magnification 19,500× (6.285 Å/pixel). Single axis tilt series were acquired bidirectionally using Serial EM (33), with a tilt increment of 2° covering -60° to +60°, a cumulative electron dose of less than 120 e^-^/Å^2^, and at a nominal defocus range between -4 and -10 µm. Intermediate magnification images were acquired to capture larger fields of view of elongated cells at a magnification of 4,800× (17.12 Å/pixel).

### Cell measurements and segmentation models

Tilt series images were motion corrected using Motioncor2 to align frames post-collection (34). Tomograms were reconstructed using fiducial-based alignments in IMOD/eTomo (35), CTF correction was carried out using the ctfplotter program in IMOD/eTomo, and the final tomograms were reconstructed by R-weighted back projection and binned by 2 or 4. Post-processing of tomograms used low pass filtering to 80 Å using IMOD or were processed using a nonlinear anisotropic diffusion filter (34). Measurements of the distance between the IM, OM, and PG layers were collected using the measurement tools in IMOD. The OM, IM, and PG layers were identified as contiguous layers surrounding the cell. The space between the IM and OM comprises the periplasmic region, which contains the PG layer that is closely associated with the OM (1). Three-dimensional rendering of cells was performed using EMAN2 neural network segmentation training (36). Between 20 and 80 boxes from each tomogram were segmented per feature (OM, IM, and extracellular vesicles) and the neural networks were trained using the recommended settings provided in the EMAN2 segmentation tutorial. Models were visualized in ChimeraX (37). Except for ChimeraX, all programs were installed and run through the chimera SBGRID infrastructure (38).

### HADA and RADA labeling

A single colony of *R. sphaeroides* was inoculated into SIS medium with appropriate antibiotics and grown overnight. Cells were then diluted to an OD600 of 0.05 in 10 mL SIS medium, with antibiotic and IPTG when appropriate, and grown to early log phase OD600 ~0.2. To the culture, a final concentration of 0.5 mM HADA (Tocris Bioscience) was added and incubated for 30 min with shaking at 30°C in the dark. Then, 1 mL of cells was pelleted and washed with PBS three times to remove excess dye, resuspended in 200 µL PBS and visualized by microscopy as described above.

For RADA (Tocris Bioscience) time-lapse, a single colony of *R. sphaeroides* was inoculated into SIS medium and grown overnight. Cultures were grown as described above. Following dilution, once cells reached early log phase OD600 ~0.2 to the culture a final concentration of 100 µM RADA was added and incubated for 1 h with shaking at 30°C in the dark. Then 1 mL of cells was pelleted and washed with SIS medium three times to remove excess dye. Cells were resuspended in 1 mL of SIS with kanamycin when appropriate. 2 µL of culture was placed inside an Ibidi slide chamber under a 1.5% agarose pad containing SIS medium and supplemented with 50 µg/mL kanamycin and 10 µM IPTG when appropriate. Cells were imaged by microscopy as described above. Each frame was taken with a 45-min interval.

## RESULTS

### Overexpression of *cenK* is sufficient to increase CenKR TCS activity *in vivo*

The CenKR TCS consists of the membrane bound sensory histidine kinase (HK) that specifically phosphorylates CenR, a cytoplasmic transcriptional response regulator (RR), in response to an unknown signal (25, 39). To expand our understanding of the function of this TCS, we examined the effects of increasing expression of either *cenR* or *cenK*. In *Caulobacter crescentus*, it was shown that overexpression of *cenR* increased the activity of this RR in a manner similar to the expression of a phosphomimetic variant, while overexpression of the HK *cenK* did not (39). Similarly, we previously observed that the introduction of a phosphomimetic *cenR* allele (D56E) into the *R. sphaeroides* genome increased the activity of this RR and produced longer cells compared to wild-type cells (25). Thus, we hypothesized that we would be able to hyperactivate this TCS in *R. sphaeroides* by overexpressing the genes encoding this system.

**Fig 1:**
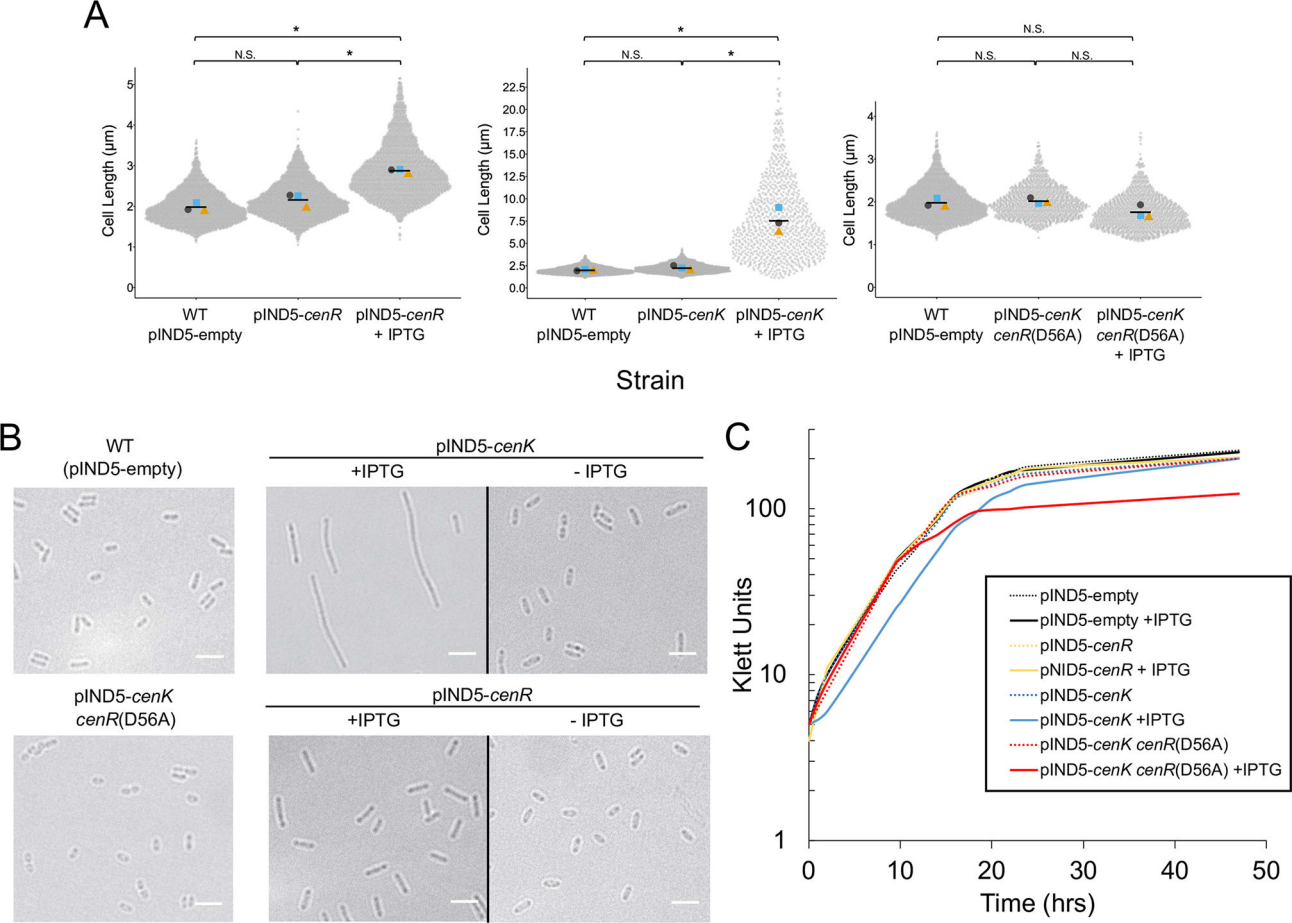
Overexpression of *cenK* increases cell length. (A) Measurements of cell length from microscopy analysis, displayed as bee swarm plots (41). Mean length values (µm) from each of the three biological replicates (grey circles, blue squares, and orange triangles) and mean length for each strain (black bar) are shown. For each biological replicate, >500 cells were analyzed. Unpaired t-tests were used to compare pooled cell length data from the mean values of each biological replicate (*n* = 3) (41). Significant *P* values <0.01 (*) are indicated by an asterisk (N.S. indicate a *P* value >0.05 and no significant difference). WT = wild type, +IPTG = ectopic expression of *cenK* induced by addition of 10 µM IPTG. (**B)** Representative bright field micrographs of each strain analyzed. Scale bar = 2 µm. (**C)** Cell density (Klett units) of *R. sphaeroides* cultures grown aerobically [1 Klett unit is equal to ~1 x 10^7^ cells/mL for WT *R. sphaeroides* (42)].

Using cell length as a known reporter of CenKR activity (25), we tested the impact of cloning either *cenR* or *cenK* into a low copy-number plasmid, pIND5, under the control of an isopropyl-β-D-thiogalactopyranoside (IPTG) inducible promoter (40). Mobilization of pIND5-*cenR* or pIND5-*cenK* into wild-type cells produced no significant change in cell length (2.16 ± 0.17 µm and 2.23 ± 0.27 µm, respectively) compared with a control strain (pIND5-empty, 1.96 ± 0.11 µm) when cells were grown in the absence of IPTG (Fig. 1A and B). However, when grown in the presence of 10 µM IPTG, cells containing pIND5-*cenR* were significantly longer (2.86 ± 0.07 µm) than the control strain (Fig. 1A and B), consistent with previous work showing phosphomimetic activation of CenR increased its function and produced cells longer than their wild-type counterparts (25). Interestingly, we found that when cells containing pIND5-*cenK* were grown in the presence of 10 µM IPTG, cells exhibited more severe morphological changes, producing cell filaments with an average length of 7.51 ± 1.40 µm (Fig. 1A and B). To test if increased expression of *cenK* was sufficient to stimulate CenK activity in a CenR-dependent manner, we analyzed the impact of a D56A amino acid substitution in CenR that has previously been shown to block phosphorylation by CenK (25). Mobilization of pIND5-*cenK* into a strain containing a *cenR*(D56A) allele in the genome produced cells with no significant difference in cell length when grown with or without IPTG compared to the control strain (Fig. 1A and B). From this, we conclude that overexpression of *cenK* is sufficient to stimulate TCS activity as phosphorylation of CenR by CenK is required to produce a cellular response and modulate transcription in *R. sphaeroides* (25).

When testing the effects of *cenR* or *cenK* overexpression on growth in the absence of IPTG, we observed no impact on either growth rates or final cell density of wild-type cells containing either pIND5-*cenR* or pIND5-*cenK* (~3 h) and a decline in generation time after IPTG was added to cells containing pIND5-*cenK* (~4 h) (Fig. 1C). Notably, there was an early cessation of growth (as measured by cell density) after IPTG is added to cells containing both the *cenR*(D56A) allele and the pIND5-*cenK* plasmid, compared to other strains analyzed (Fig. 1C). The increases in cell length and generation time observed in cells overexpressing *cenK* is similar to, but more severe than that observed in cells containing a hyperactive form of this response regulator, in a single copy that was shown to alter DNA binding and transcript levels from target genes (25). Combined, these data support the conclusion that increased activity of the CenKR TCS upon ectopic overexpression of *cenK* produces the observed changes in cell length. Given the hyper-filamentation of cells overexpressing *cenK*, we sought to gain insight into how increased CenKR activity produces these changes in cell morphology to put these observations in context with the known targets of this TCS.

### Visualizing the impact of increased CenKR activity on cell morphology

**Fig 2:**
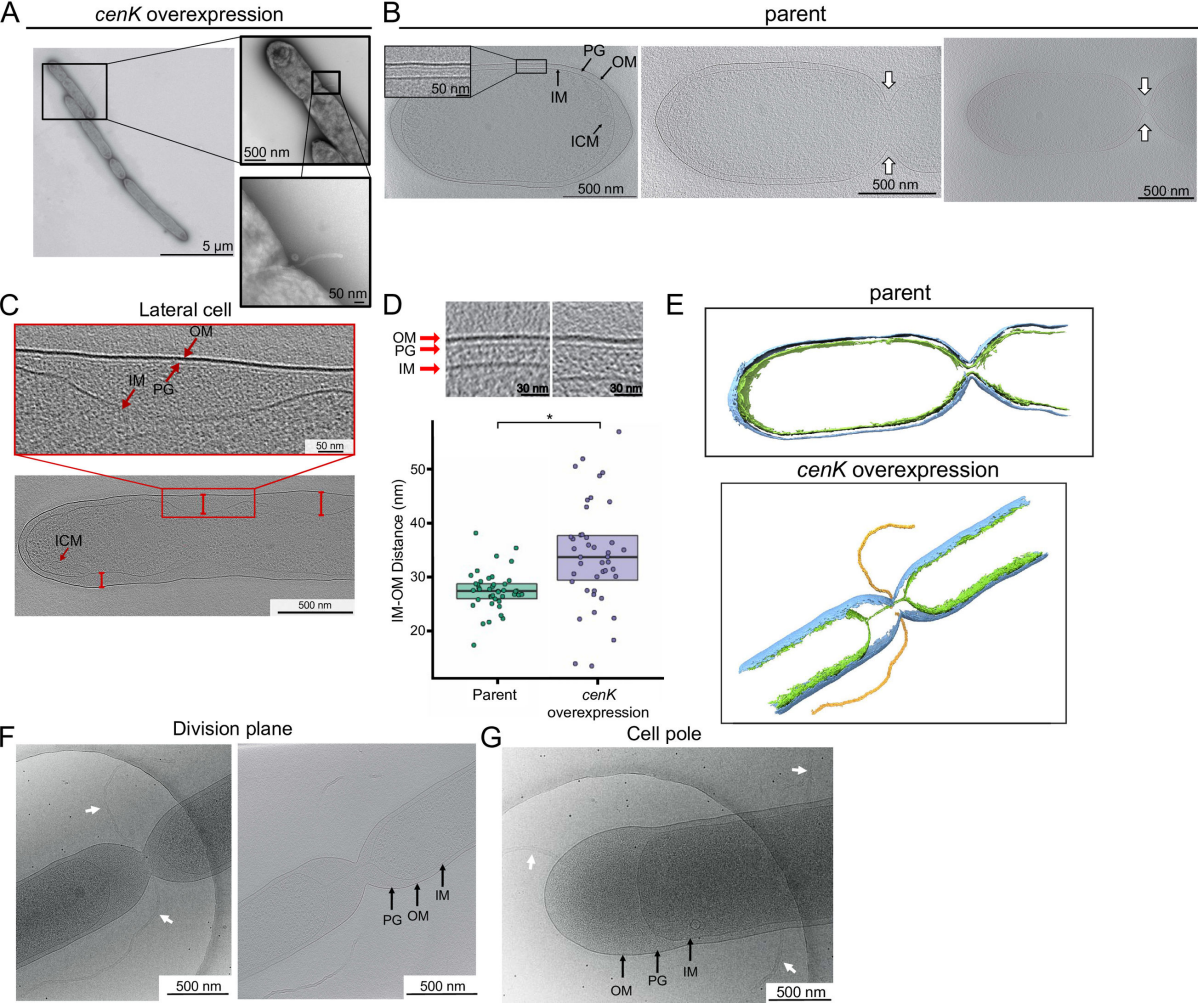
Envelope changes in cells overexpressing *cenK*. (A) TEM micrographs of negatively stained cells overexpressing *cenK*. (**B)** 3D cryo-ET reconstructions of parent cells (Δ*RSP_0382*). (Top panel) A central slice through an NAD-filtered tomographic reconstruction of the parent strain showing IM, PG cell wall, and OM. (Middle panel) A central slice through a tomographic reconstruction of the early division plane showing constriction of OM and PG layers closely associated with the IM separation. (Bottom panel) A central slice through a tomographic reconstruction of the late division plane. For tomogram slices, 10 z-slices were summed, representing a ~12.6 nm thick slice of a binned-by-2 tomogram. (**C)** Central slices through tomographic reconstructions of cells overexpressing *cenK* showing extended gaps (red lines) between the IM and OM along the length of the cell. Gaps range from ~65 to 115 nm along the cell body and from ~120 to 285 nm at the cell pole (**G**). **(D)** The periplasmic space is wider in cells with *cenK* overexpression. The distances between the layers of the cell envelope were measured using a central slice through a tomographic reconstruction of the parent strain (left image, green box plots) and *cenK* overexpression strain (right image, purple box plots). Box plots for each strain represent the median (black lines) and interquartile range (boxes) of measurements for the distance between the IM and OM or IM and PG (nm). Data points for the individual measurements are overlayed to represent the range * = *P* value < 0.01. (**E)** 3D models of parent (top) and *cenK* overexpression cells (bottom) depicting the OM (blue), IM (green), and extracellular vesicles (orange). The parent model was constructed from the right panel B. The *cenK* overexpression model represents the cell shown in panel F. (**F)** Visualization of the division plane shows separation of the IM and OM-PG layers in a 2D cryo-EM image (left). White arrows indicate a membrane protrusion from the cell division septum. A second central slice through a tomographic reconstruction of the same cell indicates a thin connection between the IMs of separating cells correlating with delayed OM constriction (right). (**G)** 2D cryo-EM image showing the impact of *cenK* overexpression on cell poles producing large periplasmic gaps between the OM and IM. Elongated membrane blebs stemming from the OM can also be seen at the pole and along the lateral cell (white arrows). For F and G tomogram slices, 10 z-slices were summed, representing a ~9.2 nm thick slice of a binned-by-2 tomogram.

Previous analysis of the CenKR regulon has shown that this TCS directly or indirectly controls the expression of genes that are required for PG synthesis, cell elongation, and division (25). Thus, we used high-resolution microscopy to visualize the impact of *cenK* overexpression on cell morphology. Negative stain electron microscopy revealed that when CenKR activity was increased, cells produced filaments and displayed evidence of cell chaining, suggesting both delayed cell division and formation of incomplete septa (Fig. 2A). We also observed surface protrusions that extended into the extracellular space and originated from these incomplete division septa (Fig. 2A) suggesting increased CenKR activity results in improper OM migration during the late stages of cell division leading to these OM blebs.

To examine the underlying morphological changes associated with increased CenKR activity, we used cryogenic electron microscopy (cryo-EM) and cryogenic electron tomography (cryo-ET) to compare each layer of the cell envelope with wild-type cells. To better visualize subcellular features and the layers of the cell envelope, we used a *R. sphaeroides* mutant that does not synthesize poly-β-hydroxybutyrate (PHB) storage granules [Δ*RSP_0382* (43)]. Our prior work has shown that PHB granules rapidly undergo damage and deformation at electron doses commonly used for cryo-ET data collection (44). In this Δ*RSP_0382* (parent) strain, 3D tomographic reconstructions revealed that *R. sphaeroides* cells divide by concatenated constriction at the midcell (Fig. 2B). During this process, the IM and OM-PG layers comigrate at division septa and remain tightly associated throughout cytokinesis and IM fission (Fig. 2B and E top panel).

In contrast, cryo-EM images and 3D tomograms of cells overexpressing *cenK* in a Δ*RSP_0382* background revealed an increased distance between the IM and OM over the entire length of the cell and at the cell poles. Additionally, these morphological changes were found in cells at different phases of the cell cycle (Fig. 2C–G; Fig. S1 and S2). We also observed instances of differential spacing between the IM and OM along the length of the cell that produced a repetitive, wave-like pattern (Fig. 2C). Analysis of tomographic reconstructions of these data predict that changes in intramembrane spacing specifically affect the IM, while the properties of the OM and PG layers remain relatively unchanged in cells with increased CenKR activity (Fig. 2C). Measurements of the distance between the IM and OM or IM and PG layers [excluding the regions of “abnormal”, extended periplasmic width (Fig. 2C, red bars)] indicates that the average width of the periplasmic space in the cells overexpressing *cenK* is significantly increased along the length of the cell (33.9 ± 4.8 nm) relative to that measured in the parent cells (27.3 ± 1.8 nm) (Fig. 2D). In contrast, the distance between the OM and PG layer measured from these images were not found to be significantly different in cells overexpressing *cenK* when compared to the control strain (Fig. S1A–C).

In cells with increased CenKR activity, we found evidence of cells with multiple division septa (75%; *n* = 85/113, Fig. S1C), as well as asymmetric division (Fig. S1D; Fig. S2A and B ), that were not observed in the parental strain (Fig. S2C). These multiple septa could be separated into two classes: those that resembled normal division sites (Fig. 2B, Video S3 at doi.org/10.6084/m9.figshare.22280611) where the IM and OM comigrate and are “connected” during constriction (Fig. S1D and E), and those where the OM failed to invaginate contiguously with the IM, suggesting a stalled division process (Fig. 2F and E bottom panel, Video S4 at doi.org/10.6084/m9.figshare.22280617). In these latter instances, the IM and OM-PG layers appear to be disconnected during cell septation resulting in an extended periplasmic space. The reconstructed images provide evidence that the IM appeared to complete fission and was dissociated from the OM, resulting in an increased distance between the cytoplasmic compartments of the resulting daughter cells (Fig. 2F), compared to the control strain where the IM, PG, and OM are tightly associated throughout cell division (Fig. 2B middle and right panels). For these abnormal division sites, changing the Z-plane of these tomographic reconstructions revealed a thin, continued interconnection between the IMs of daughter cells, suggesting that the normal process of cytokinesis was also disrupted in cells that have increased CenKR activity (Fig. 2F right panel, Fig. 2E bottom panel, and Video S4 at doi.org/10.6084/m9.figshare.22280617). In some reconstructions, there is evidence for more subtle instances of this IM-OM uncoupling, with evidence of extended periplasmic regions immediately adjacent to the division septum (Fig. S1F). This separation of the IM and OM, either at the division septum, along the length of the cell or at the cell poles was observed in ~21% (*n* = 18/85) of the cells with increased CenKR activity that were imaged by Cryo-EM (Fig. S1C).

In most of these tomographic reconstructions, we observed protrusions at the sites of IM fission (Fig. 2F), the cell poles (Fig. 2G), and/or along the length of cells with increased CenKR activity (Fig. S3) that were not present in reconstructions of the parent strain (Fig. 2B), or observed in previous analyses of wild-type *R. sphaeroides* cells (45 - 49). In reconstructions of cells with OM protrusions, we clearly resolved the extracellular polysaccharide (EPS) coat along the exterior of cell and surrounding membrane blebs/vesicles (Fig. S3; Video S5 at doi.org/10.6084/m9.figshare.22280614). This complements our prior structural studies of outer membrane vesicles (OMVs) from *Vibrio vulnificus* where the capsular polysaccharide is retained on the outer surface (OM) of the cells and OMVs (50). When we constructed segmentation models from these tomograms (Fig. 2E bottom panel, S1D, S3; Video S4 at doi.org/10.6084 /m9.figshare.22280617; and Video S5 at doi.org/10.6084 /m9.figshare.22280614), they also indicated that the OM was the origin of these protrusions. We also observed differences in inner cytoplasmic membrane vesicles (ICMs) produced by parent (Fig. 2B) and *cenK* overexpression strains (Fig. 2C; Fig. S3E). These ICM vesicles play a role in anaerobic photosynthesis, housing photosynthetic machinery (51) and, in *R. sphaeroides*, are observed as circular vesicles that bud from the IM into the cytoplasm (47 - 52 - 52). However, in cells overexpressing *cenK* these ICM vesicles appear elongated and irregularly shaped compared to parent cells suggesting the normal synthesis of these vesicles is also disrupted in this strain. Together, these data lead us to propose that cells which overexpress *cenK* have altered interactions between the OM, PG cell wall, and surface components resulting in severe division defects and formation of OM protrusions. Further, these disruptions affect overall cell envelope homeostasis leading to defects in other normal cell envelope remodeling events (i.e., ICM formation, elongation, and cytokinesis). Because of these findings, we performed experiments to provide more insight into the observed morphological changes in cells that have increased CenKR activity.

### Increased CenKR activity leads to mislocalization of the Tol-Pal complex

**Fig 3:**
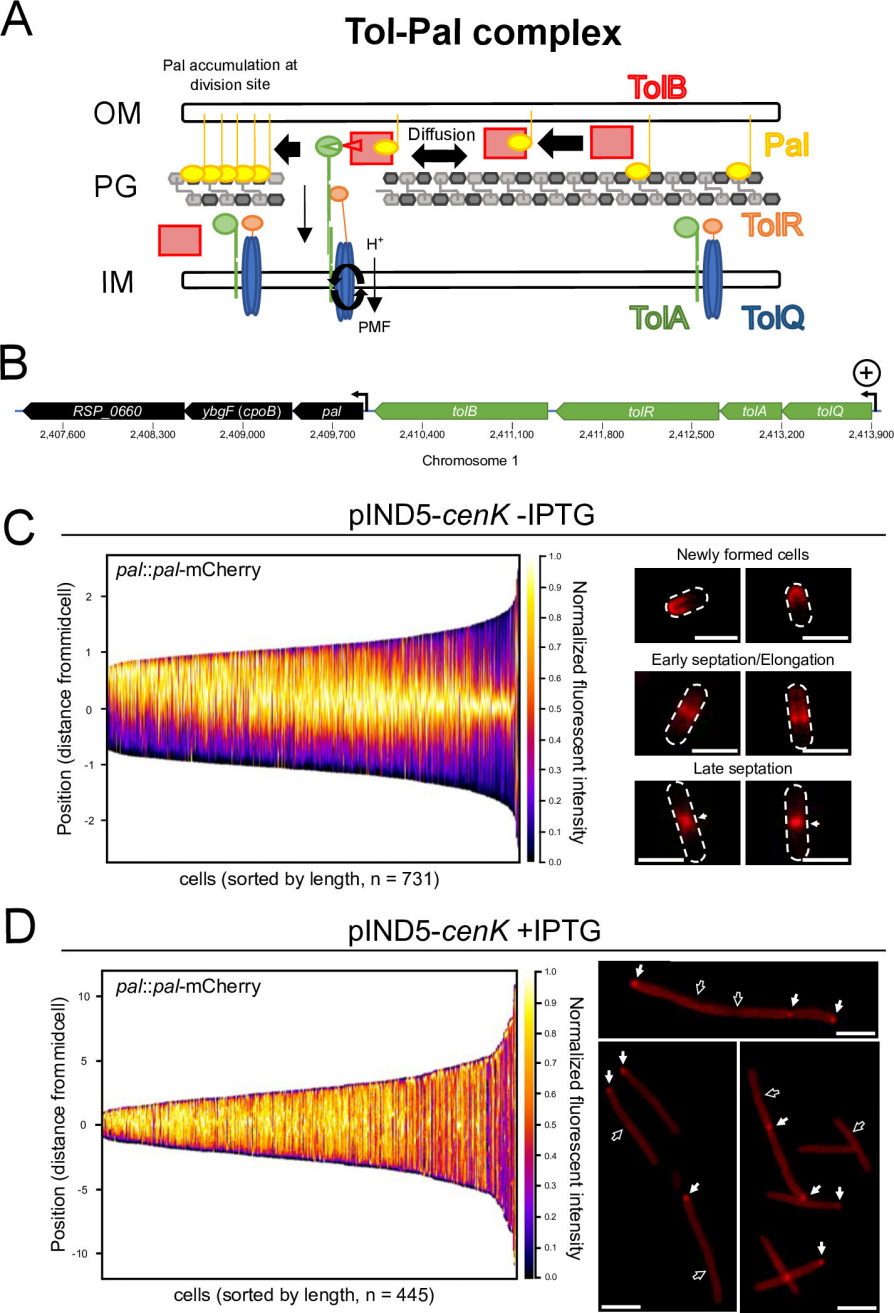
Overexpression of *cenK* affects the topological localization of the Pal OM lipoprotein. (**A)** Schematic of Tol-Pal OM complex function (10). The interaction of Pal (yellow) with PG and its diffusion through the periplasm is facilitated by interactions with TolB (red). During cell division, recruitment of TolQRA to the midcell sequesters Pal to the septum. (**B)** The *tol* and *pal* operons. The *tolQRAB* genes and *pal-cpoB-RSP_0660* are in two transcriptional units in *R. sphaeroides*, with known transcriptional start sites indicated (53). Expression of the transcriptional unit containing *tolQRAB* (green) is increased by CenKR activity . (**C)** Pal-mCherry spatial localization during the cell cycle as visualized by fluorescence microscopy. (Left) Demographs of Pal-mCherry displaying normalized fluorescence intensity profiled over the length of wild-type cells (y-axis). Normalized intensity profiles for each cell were combined and sorted (from left to right) by length to model growth and division throughout the cell cycle (x-axis). Cells were also sorted from top to bottom orienting the pole with the most fluorescence at the top (y-axis). (Right) Representative micrographs with a dotted line showing outlines of the shape of cells, scale bar = 2 µm. Accumulation of Pal-mCherry at the division plane is indicated by arrows. (**D)** Pal-mCherry localization when cells containing pIND5-*cenR* are grown in the presence of 10 µM IPTG. (Left) Demograph displaying Pal-mCherry normalized fluorescence intensity profiles. Cells were sorted from left to right by length and top to bottom orienting the pole with the most fluorescence at the top. (Right) Representative micrographs of cells overexpressing *cenK*, white arrows indicate accumulation of Pal at cell poles and stalled division septum, empty arrows indicate apparent division sites lacking Pal, scale bar = 2 µm.

The previous results suggest that overexpression of *cenK* affects the completion of cytokinesis leading to stalled cell division and cell chaining (Fig. 2; Fig. S1–S3). From previous analyses (25), we know that the RR CenR binds to and activates the expression of the *tolQRAB* genes that encode subunits of the Tol-Pal complex but does not result in a significant change in the transcript levels of the downstream *pal-cpoB-RSP_0660* transcriptional unit (Fig. 3A and B). We hypothesized that increased expression of the *tolQRAB* genes in cells with increased CenKR activity leads to elevated levels of the TolQRA subunits of the divisome and mislocalization of Pal affecting the normal function of the Tol-Pal complex.

To test this hypothesis, we compared Pal localization in wild-type and *cenK* overexpression strains (Fig. 3C and D). To do this, we fused the gene encoding a mCherry reporter to the Pal coding region at the native locus (*pal::pal-mCherry*), generating the C-terminal translational fusion, Pal-mCherry. Using this reporter, we observed fluorescence of the Pal-mCherry protein in wild-type cells (Fig. S4) and strains containing the *cenK* overexpression plasmid in the absence or presence of IPTG (Fig. 3C and D). Wild-type cells containing this Pal-mCherry construct exhibited no observable filamentation, chaining, or other cell envelope phenotypes (Fig. S4A and B) suggesting the presence of this Pal-mCherry translational fusion had no major impact on Tol-Pal activity or function.

To analyze the intracellular localization of Pal-mCherry in these strains throughout the cell cycle, we assembled normalized fluorescence profiles along the length of all individual cells. Sorting cell populations by length allowed us to visualize cell cycle dynamics, using the assumption that cell length is a suitable indicator of cell cycle progression (54). During the elongation phase in wild-type cells, Pal-mCherry fluorescence was found to be dispersed between the new cell pole and the midcell while being mostly excluded from the old cell pole. As cells elongate, Pal-mCherry accumulates at the midcell likely coinciding with early septum formation. During division, Pal-mCherry fluorescence is focused at the midcell before diffusing along the half-cell (between the new pole and midcell) for a new phase of elongation in the daughter cell (Fig. 3C; Fig. S4). Similar pole-to-midcell localization dynamics have been observed in other Gram-negative bacteria (8, 9, 14, 15) suggesting that the pattern of cell cycle Tol-Pal localization is conserved in *R. sphaeroides*.

When we monitored the localization of Pal-mCherry in cells with increased CenKR activity (Fig. 3D), we observed a different pattern of fluorescence. In this strain, Pal-mCherry was more diffuse throughout the length of the cell and a significant fraction of the Pal-mCherry protein was retained at the poles of the filamentous cells (Fig. 3D, white arrows). We also found that ~60% of the apparent division septa in cells with increased CenKR activity lacked evidence for the accumulation of Pal-mCherry fluorescence (Fig. 3D, empty arrows). To test if the dispersion of Pal-mCherry fluorescence was due to increased expression of *pal-mCherry* from the native *pal* locus, we measured total cellular fluorescence (Fig. S4C). We observed no significant difference in strains with increased or basal *cenK* expression. As a control, we analyzed a strain ectopically expressing (pIND5-*pal-mCherry*), finding a significant increase in cellular fluorescence when *pal-mCherry* was overexpressed ectopically compared to *cenK* basal and overexpression strains (Fig. S4C). Regardless, cells overexpressing *pal-mCherry* displayed similar cell cycle localization dynamics as seen in wild-type cells (Fig. S3D), suggesting that increased Pal-mCherry abundance alone is not sufficient to disrupt pole-to-midcell diffusion.

Taken together, these data suggest that the division defect in cells containing increased CenKR activity is associated with a failure to move Pal away from the poles of newly formed cells and relocalize it to septa during the cell cycle. The demograph for cells containing increased CenKR activity supports the observation that Pal-mCherry fluorescence is widely distributed throughout the cell envelope and further suggests a reduced rate of Pal diffusion during the cell cycle in these filaments. Finally, these data are consistent with previous data indicating that expression of the *pal-cpoB-RSP_0660* locus is not regulated by CenKR activity (25) and suggest that overexpression of Pal is not sufficient to alter localization dynamics (Fig. S4D). Instead, the changes in Pal localization are likely driven by increased expression of *tolQRAB* (Fig. 3A and B). Given the importance of Pal localization for proper cell division and the previously described phenotypes, we asked if the function of additional components of the cell elongation or division machinery was affected in cells with increased CenKR activity.

### Increased CenKR activity alters PG dynamics

It is also known that the CenKR TCS increases the expression of genes encoding members of the elongasome, such as MreB, MreC, RodZ, and PBP2 (25). Rod-shaped bacteria like *R. sphaeroides* often utilize the cytoskeletal homolog MreB to coordinate the localization of the elongation-specific transpeptidase (TPase) penicillin-binding protein 2 (PBP2) in order to maintain a rod shape (7, 55). Given the changes in gene expression and cell length observed when *cenK* is overexpressed, we hypothesized there were changes in PG deposition, cross-linking, and/or hydrolysis in this strain compared with wild-type cells.

**Fig 4:**
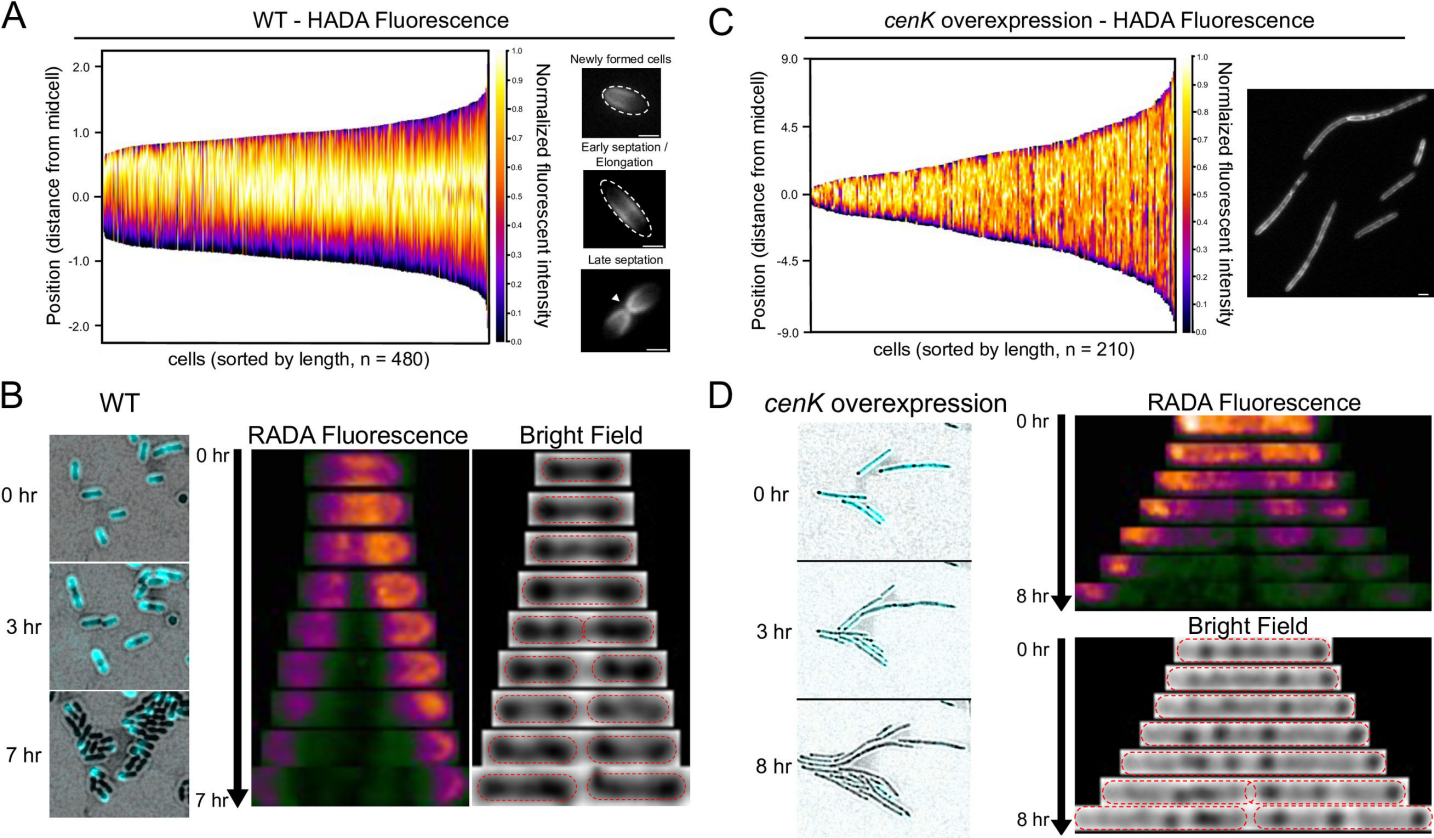
Spatial and temporal PG biosynthesis in *R. sphaeroides* wild-type and cells overexpressing *cenK*. (A, B). Demographs of HADA-normalized fluorescence intensity profiled over cell length (y-axis). The normalized intensity profile for each cell was combined and sorted by length to model growth and division throughout the cell cycle (x-axis) and from top to bottom to orient the pole with the most fluorescence at the top (y-axis). Representative micrographs of WT cells (**A**) those overexpressing *cenK* (**B**), dotted lines outline the shape of cells, and white arrow indicates division septum, scale bar = 2 µm. (**B, D)** Time-lapse microscopy recording fluorescent D-amino acid (FDAA) RADA incorporation (pulse) and dispersion due to nascent PG synthesis (chase) over several cell cycles in WT cells (**B**) and cells overexpressing *cenK* (**D**). (Left) Micrographs with merged bright field and RADA (cyan color scale) channels of a field taken at different times during growth. (Right) Time-lapse pictures of growth. RADA fluorescence (in plasma color scale) and complementary bright field channels of a single cell are shown (dotted red outline of cell shape).

To test this hypothesis, we compared PG assembly in wild-type cells to those with increased CenKR activity. In one approach, we monitored PG biosynthesis/remodeling by growing cells in the presence of HADA (a fluorescent hydroxycoumarin moiety linked to 3-amino-D-alanine) that can be incorporated into PG via periplasmic enzymes (TPases) (56, 57). Using the assumption that cell length is a suitable indicator of cell cycle progression (54), we analyzed the pattern of fluorescence in a population of exponentially growing wild-type cells after a 30 min (1/5th doubling time) “short-pulse” with HADA (Fig. 4A). We observed that HADA fluorescence in newly formed (shorter length) cells is localized to one pole. As cells elongate, this fluorescence shifts to the midcell and is concentrated at the division septum, likely representing nascent PG synthesis/remodeling required for midcell constriction. Following division, HADA fluorescence persists at the newly formed cell poles, and the cell cycle beings again. These data are consistent with previous analyses of HADA incorporation in wild-type *R. sphaeroides* (27). However, it is known that the incorporation of HADA reports on the periplasmic activity of D,D-TPases (PBPs) and L,D-TPases that add this molecule into the tetrapeptide stems of PG (57). Therefore, we hypothesized that the observed fluorescence within the newly formed cell poles may represent residual/transient activity of PG cross-linking or other remodeling enzymes rather than nascent polar PG synthesis that occurs in other α-proteobacteria (commonly *Rhizobiales*) (58).

To test this hypothesis, we used a second approach to visualize, in real time, the sites of PG synthesis by native cytoplasmic precursor molecules. To do this, we analyzed time-dependent RADA (5-carboxytetramethylrhodamine conjugated to 3-amino-D-alanine) fluorescence (56, 57). Treatment of exponentially growing wild-type cells with RADA led to uniform PG labeling (Fig. 4B). By monitoring growth of these labeled cells after removing unincorporated RADA (chase), we observed loss of fluorescence at the midcell expanding toward the poles as a function of time (Video S1). Following cell division, the “old” poles of each daughter cell retained RADA fluorescence with new PG synthesis (as indicated by loss of RADA fluorescence) appearing to initiate from the new midcells (Fig. 4B). Combined, these data suggest that wild-type *R. sphaeroides* exclusively uses medial PG synthesis for elongation throughout the cell cycle (Fig. 4B), and the fluorescence observed at the poles during a short-pulse of HADA (Fig. 4A) represent residual activity of PG remodeling rather than nascent synthesis.

When we performed similar analyses in cells overexpressing *cenK*, we observed a different pattern of PG synthesis/remodeling and spatial cycling when using HADA as a reporter (Fig. 4C). In this strain, the entire length of the cells showed HADA fluorescence, suggesting that cells with increased CenKR activity are synthesizing/remodeling PG throughout the cell. Indeed, the observed pattern of HADA fluorescence predicts PG synthesis/remodeling at the multiple sites in the filamentous and chained cells (Fig. 4C, right panel). Examining synthesis of PG as a function of time in cells overexpressing *cenK* by monitoring RADA fluorescence via pulse-chase (Fig. 4D), we observed broad incorporation of this reporter throughout the length of cells with increased CenKR activity (pulse). During the chase period (after RADA was removed), fluorescence was lost from multiple sites concurrently suggesting cells with increased CenKR activity initiates, synthesizes, and assembles PG from multiple locations. We also observed a loss of RADA fluorescence throughout the length of cells with increased CenKR activity over the course of the analysis (Video S2). We propose that the observed loss of RADA fluorescence reflects increased activity of PG carboxypeptidases or hydrolases that are known CenKR target genes (25) and have been reported to promote PG turnover in other bacteria (57, 59).

### Cells with increased CenKR activity have increased sensitivity to PG-active chemicals

**Fig 5:**
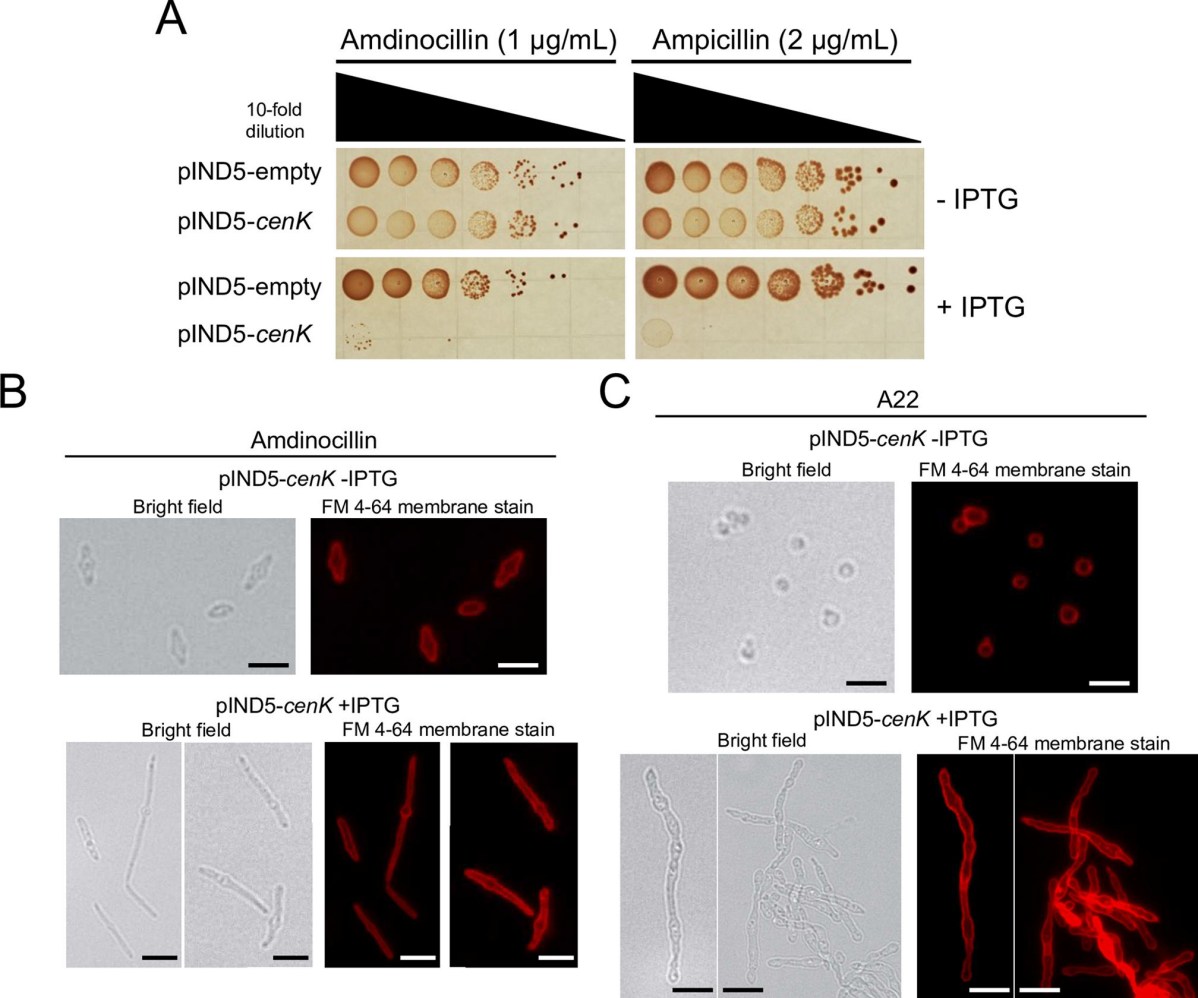
Antibiotic treatment of *R. sphaeroides* strains. (A) Cells containing pIND5-*cenK* were tested for sensitivity to β-lactam antibiotics in the presence or absence of IPTG. (**B)** Antibiotic treatment of cells with amdinocillin. Bright field and fluorescence micrographs of cells uninduced (−IPTG, Top) and induced (+IPTG, Bottom) for ectopic expression of *cenK*. Scale bar = 2 µm. Amdinocillin (0.5 µg/mL). (**C)** Antibiotic treatment of cells with A22. Bright field and fluorescence micrographs of cells uninduced (−IPTG, Top) and induced (+IPTG, Bottom) for ectopic expression of *cenK*. Scale bar = 2 µm. A22 (10 µg/mL).

It has been shown that loss of CenKR activity increases sensitivity to β-lactam antibiotics that block nascent PG synthesis and detergents that permeabilize the OM (45). Given the observed pattern of PG biosynthesis in cells with increased CenKR activity, we asked if these cells also have altered sensitivity to β-lactam antibiotics. To do this, serial dilutions of cells containing the pIND5-*cenK* plasmid were spotted onto plates containing amdinocillin [also known as mecillinam, which specifically inhibits PBP2 in *R. sphaeroides* (27, 60, 61)] or ampicillin [which inhibits several PBPs (62)] in the presence (+) or absence (−) of IPTG (Fig. 5A). We found that cells induced for *cenK* expression by growth in the presence of IPTG were more sensitive to either antibiotic compared to control cells or when cells containing the *cenK* plasmid were grown in the absence of IPTG (Fig. 5A). Most Gram-negative bacteria are naturally resistant to PG active compounds like β-lactam antibiotics that are excluded by the OM (1). Therefore, increased sensitivity to these compounds by cells containing increased CenKR activity predicts that these cells have defects in OM integrity.

Previously, it has been shown that inhibiting the activity of PBP2 in *R. sphaeroides* results in midcell swelling consistent with defects in elongation-specific PG synthesis in this organism (27, 60, 61) and other Gram-negative bacteria (63, 64). Given the observed pattern of PG synthesis and remodeling, we assayed the impact of inhibiting PBP2 activity in cells with basal and increased CenKR activity. Addition of subinhibitory concentrations of amdinocillin to cells containing pIND5-*cenK* in the absence of IPTG resulted in midcell swelling (Fig. 5B, top panels) consistent with previous analysis of wild-type cells (27, 60, 61). Treatment of this strain with amdinocillin in the presence of IPTG produced filamentous cells, as expected, and showed evidence for midcell swelling (Fig. 5B, bottom panels). In the presence of amdinocillin, we observed only one region of swelling per cell in cells with increased CenKR activity. Combining these results with those from the analysis of PG synthesis suggests that while cells with increased CenKR activity appear to initiate PG assembly from several sites (Fig. 4D), PBP2 activity and localization are not required for this behavior.

Others have proposed that swelling of wild-type cells treated with amdinocillin was correlated with the localization of the cytoskeletal protein MreB directing PG synthesis at the elongasome in *R. sphaeroides* (61). Based on this, we tested the impact of treatment with A22, which blocks polymerization of MreB filaments in *R. sphaeroides* (27) and other Gram-negative bacteria (65, 66), on cells with and without increased CenKR activity. We found that treatment of cells containing pIND5-*cenK* with A22 in the absence of IPTG (Fig. 5C, top panels) produced small, round cells that cannot elongate to form a normal rod shape, consistent with published analysis of wild-type cells (27). However, treatment of the cells containing pIND5-*cenK* that were grown in the presence of IPTG with A22, led to filamentous cells with irregular widths and multiple sites of swelling (Fig. 5C, bottom panels). The response of cells with increased CenKR activity to amdinocillin and A22 suggests that PBP2 does not colocalize with MreB in cells overexpressing *cenK*. Furthermore, these data predict that, in cells with increased CenKR activity, MreB polymerization occurs throughout the length of the filaments and synthesize PG independent of the elongasome.

### Increased CenKR activity alters MreB localization

**Fig 6:**
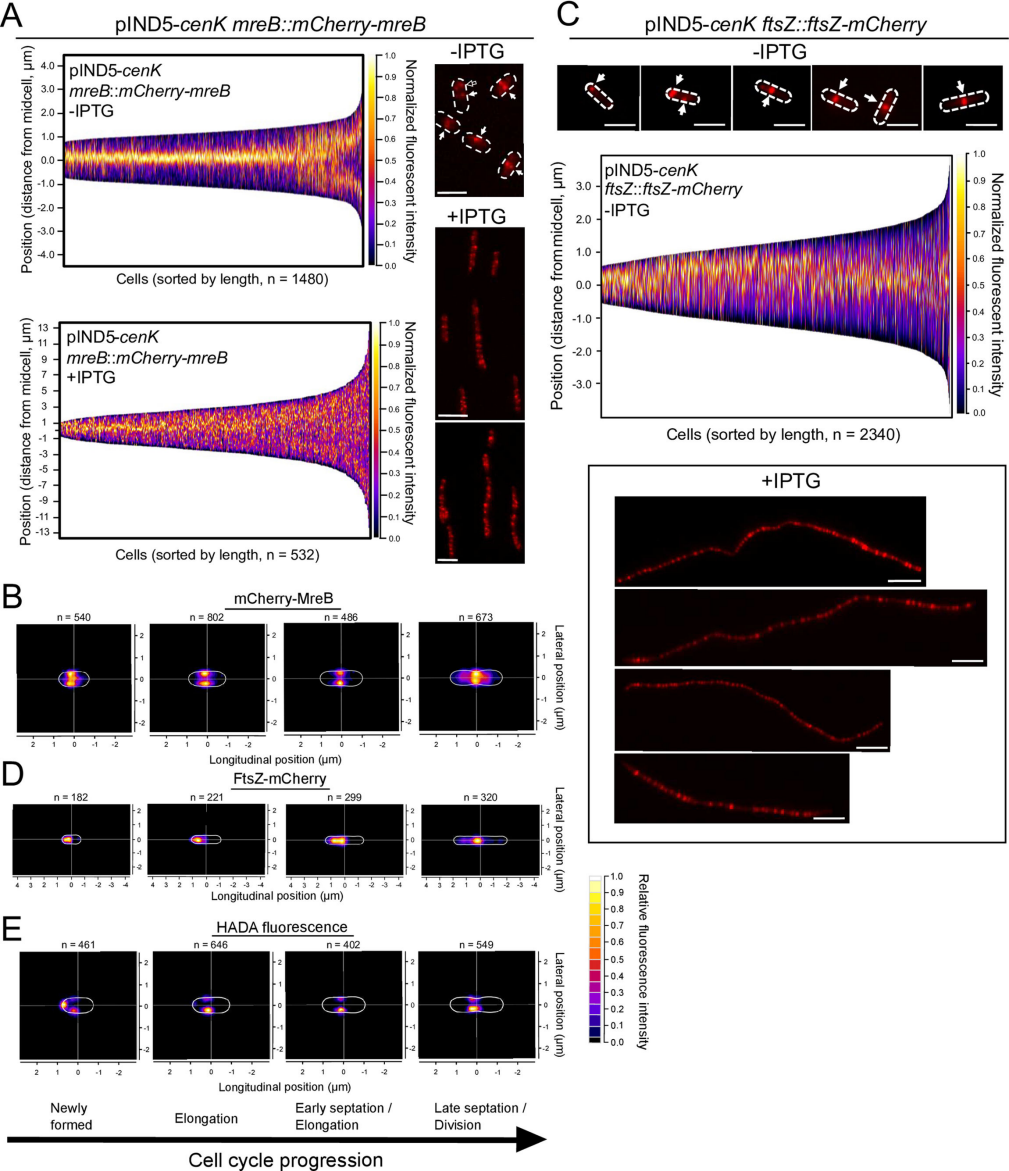
Accumulation and localization of MreB and FtsZ in cells overexpressing *cenK*. (A) Demographs of mCherry-MreB normalized fluorescence intensity profiled over cell length (y-axis) in cells with basal (−IPTG, top left) or increased (+IPTG, bottom left) *cenK* expression. The normalized intensity profile for each cell was combined, and cells were sorted by length to model growth and division throughout the cell cycle (x-axis) and arranged top to bottom to orient the pole with the most fluorescence at the top (y-axis). Accompanying fluorescence microscopy images (right panels) of mCherry-MreB localization in cells with basal (−IPTG, top right) or increased (+IPTG, bottom right) *cenK* expression. Dotted lines indicate cell boundaries, white arrows indicate location of mCherry-MreB foci at the apparent midcell, empty arrows indicate mCherry-MreB foci at apparent quarter-cell, scale bar = 2 µm. (B, D, E) Density heat maps of fluorescence maxima of mCherry-MreB (**B**), FtsZ-mCherry (**D**), or HADA (**E**) projected on cell map (white line). Cells were binned into four categories based on cell length to simulate cell cycle progression (the shortest cells on the left to the longest on the right). Representative average cell perimeters are displayed by the white boundary lines for each plot and category. (**C)** Fluorescence microscopy images of FtsZ-mCherry localization in cells uninduced (−IPTG, top panels) and induced (+IPTG, bottom panels) for ectopic expression of *cenK*. Dotted lines indicate cell boundaries, and white arrows indicate FtsZ-mCherry foci, scale bar = 2 µm. (Middle panel) Demographs of FtsZ-mCherry normalized fluorescence intensity profiled over cell length (y-axis). The normalized intensity profile for each cell was combined, and cells were sorted by length to model growth and division throughout the cell cycle (x-axis) and arranged top to bottom to orient the pole with the most fluorescence at the top (y-axis).

In many bacteria, MreB is an integral component of the elongasome that serves to localize and coordinate PG synthesis during cell elongation (3). Given the observed pattern of PG synthesis and the cell swelling produced when cells are treated with A22, we tested if increased CenKR activity affected MreB localization. To do this, we monitored the cellular position of a translational fusion of mCherry-MreB expressed from the native *mreB* locus (*mreB::mCherry-mreB*). Analysis of cells containing this mCherry-MreB fusion showed foci of fluorescence positioned at the midcell (Fig. 6A, white arrows) or quarter cell (Fig. 6A, empty arrow). Analysis of the mCherry-MreB fluorescence pattern in uninduced cells suggest that MreB remains at the midcell until early septum formation after which it moves to the quarter cell (representing the midcell of the forming daughter cells) shortly before division/midcell constriction (Fig. 6A top panel, 6B), confirming previous predictions of MreB localization in *R. sphaeroides* (60, 61).

In cells with increased CenKR activity, we observed mCherry-MreB fluorescence at numerous locations along the length of the cell filament (Fig. 6A, bottom panels), and we often failed to observe a pattern of opposing foci at the midcell like those seen in cells in the absence of IPTG (Fig. 6A bottom panels) and previously in wild-type cells (50, 51). Instead, the pattern of mCherry-MreB fluorescence along the entire length of cells with increased CenKR activity (1.04 ± 0.36 foci/µm, *n* = 671) compared to cells with basal CenKR activity (0.65 ± 0.35 µm, *n* = 1,739) (Fig. S5A and S5B) predicts that the organization of MreB filaments is mislocalized, providing a likely explanation for the unusual pattern of PG synthesis, initiation, and turnover that was observed in this strain.

### FtsZ localization is altered in cells with increased CenKR activity

In *E. coli*, the assembly and subcellular position of MreB filaments is proposed to be dependent on the tubulin homolog FtsZ during septation (67 - 70). Our data and the published cell cycle localization pattern of *R. sphaeroides* MreB suggest there is also a cooperative relationship between the elongasome and divisome in this bacterium (71). This, plus the cell division and Pal localization defects observed in cells overexpressing *cenK* prompted us to compare FtsZ localization in cells with basal and increased CenKR activity.

In cells with basal CenKR activity (−IPTG), FtsZ-mCherry fluorescence was observed at the new pole of recently formed daughter cells (Fig. 6C, top panels). During cell elongation, FtsZ fluorescence shifts to the midcell at the early stages of septum formation, a pattern that persists throughout cytokinesis and late stages of cell division (Fig. 6C top panels, Fig. 6D). This pattern of fluorescence supports the conclusion of dynamic positioning of FtsZ throughout the cell cycle in *R. sphaeroides* (Fig. 6D), as previously predicted from analysis of a FtsZ-YFP translational fusion that was ectopically expressed from a plasmid (71, 72). In contrast, analysis of FtsZ-mCherry localization in cells overexpressing *cenK* showed fluorescence that was present at intervals along the length of the cell (Fig. 6C, right panels). In cells overexpressing *cenK,* we observed 0.8 ± 0.15 foci/µm (*n* = 79) of FtsZ-mCherry compared to 0.6 ± 0.3 foci/µm (*n* = 1,610) in cells with basal *cenK* activity (Fig. S5C and D). These data suggest that FtsZ rings, while more abundant in cells overexpressing *cenK*, appear to maintain regular spacing in this strain. However, this conclusion is likely confounded by the additive effect on cell filamentation in strains overexpressing *cenK* in the presence of the FtsZ-mCherry translational fusion as these cells are even longer than those overexpressing *cenK* alone (Fig. 1B vs Fig. S5D). Therefore, any toxicity resulting for the genomic integration of the FtsZ-mCherry fusion is significantly increased by hyperactivation of the CenKR TCS. In any case, these data support the conclusion that increasing CenKR activity results in delayed division resulting in the accumulation of FtsZ rings along the length of the cell.

## DISCUSSION

**Fig 7:**
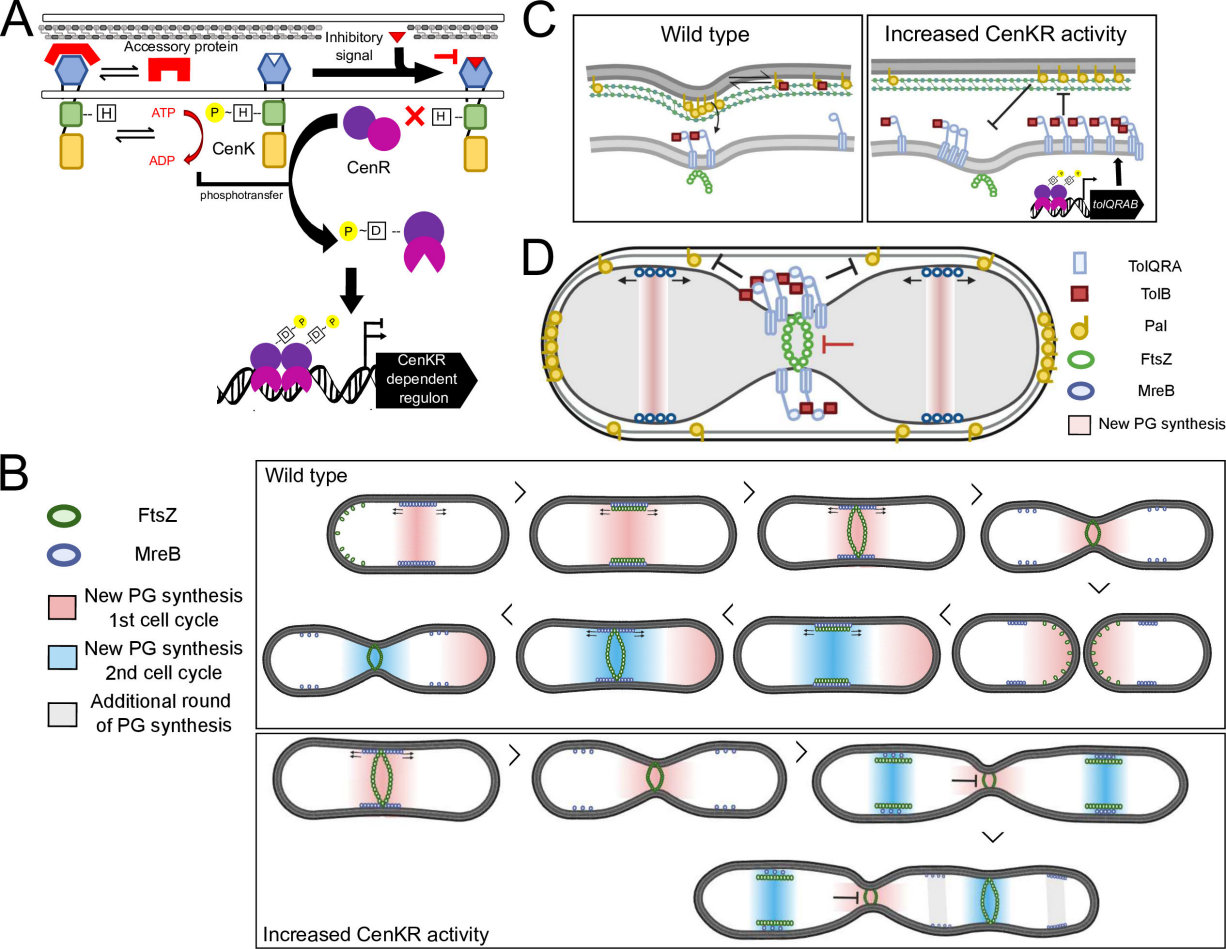
Model for how CenKR activity impacts PG biosynthesis and cell division machinery in *R. sphaeroides*. (A) A putative and unknown cell envelope derived ligand controls CenK activity. Alternatively, the activity of CenK may be modulated by a periplasmic accessory protein (see Discussion). (**B)** Localization of PG biosynthesis, MreB, and FtsZ throughout the cell cycle. Increased CenKR activity results in “locked” Z-rings that accumulate along the length of the cell, while MreB continues to polymerize and direct PG synthesis. (**C)** Wild-type levels of TolQRA-B allow proper mobilization and captureof Pal at division septum. Accumulation of TolQRA-B due to increased CenKR activity results in buildup of these complexes throughout the cell thereby reducing Pal mobility and preventing accumulation at division septum. (**D)** A model to explain the presence of a stalled division septum in cells overexpressing *cenK*. In cells with increased CenKR activity, the elevated levels of TolQRAB alter interactions between the IM TolQRA complex and periplasmic TolB, thereby preventing diffusion of Pal throughout the cell length. This interaction between TolQRA and TolB prevents Pal accumulation at division septum leading to disassociation of the IM and OM-PG layers and incomplete division. Thus, FtsZ is unable to disassemble from the divisome, while MreB migrates to the newly formed midcells and begins another cycle of elongation as in (**B**).

This work sought to gain additional insight into how changes in the activity of the essential CenKR TCS affect cell morphology in *R. sphaeroides* (25). Specifically, we focused on analyzing changes in the positioning of key cell elongation and division machinery since the CenKR TCS was previously shown to regulate transcription of many genes that are involved in these systems. Below, we summarize the major new information that has been obtained by analyzing the impact of increased *R. sphaeroides* CenKR activity on cell elongation and division. We also present a working model for how overexpression of *cenK* impacts cell shape, elongation, and division in *R. sphaeroides* (Fig. 7), and compare the behavior of this α-proteobacterium to what is known in other Gram-negative bacteria. Given the conservation of CenKR and its predicted target genes across α-proteobacteria, we expect that major concepts gleaned from analyzing *R. sphaeroides* will be translatable to other bacterial species.

### Overexpression of *cenK* is sufficient to increase CenKR TCS activity

The properties of cells ectopically expressing *cenK* support the conclusion that this was sufficient to increase activity of this TCS in a CenR-dependent manner (Fig. 1). This observation reveals a somewhat unusual feature of the CenKR TCS since overexpression of a HK often has no impact or lowers activity of a TCS in the absence or presence of an activating stimulus (73 - 75). Rather, canonical TCS are typically sensitive to the levels of RR (75), which is consistent with our observations, and is likely due to the known binding of non-phosphorylated CenR (25) and other RRs (76) to promoter sequences or transient phosphorylation of RRs by small molecule donors (77). Since overexpression of *cenK* is sufficient to stimulate TCS activity (Fig. 1), we propose that the activity of CenK is regulated by an unknown inhibitory signal in *R. sphaeroides* (Fig. 7A), as has been reported for some other TCSs (78 - 80) including ones that control cell envelope processes in other bacteria (81, 82). Moreover, we propose that the basal, and likely constitutive, activity of CenKR contributes to the essentiality of this TCS in other α-proteobacteria (25, 39, 83).

CenKR is a direct transcriptional regulator of genes that encode proteins needed for normal cell elongation and division, including subunits of the Tol-Pal complex (Fig. 3B), and cell wall biosynthesis/remodeling genes (25). Therefore, it is possible that CenKR responds to a signal that is derived from a normal or abnormal temporal cell cycle process. While overexpression of *cenK* is sufficient to stimulate TCS activity in *R. sphaeroides*, this is not the case in *C. crescentus*, another α-proteobacterium where CenKR activity has been studied (39). Furthermore, while the essentiality of *cenR* appears to be widely conserved across α-proteobacteria, this is not the case for *cenK* that is essential in some, but not all species (25). We speculate that the predicted differences in signal recognition and its effect on TCS activity likely stem from the different modes of PG synthesis and cell division, which culminate in vast differences in cell morphology, dimorphism, and prosthecae synthesis throughout α-proteobacteria (4). Additional work is needed to identify the signal ligand(s) or possible protein interaction(s) that controls CenKR activity in *R. sphaeroides* and how it may differ among species to better understand the regulatory, activity, and essentiality differences of this TCS in α-proteobacteria.

### The process of cell elongation and division in *R. sphaeroides*

Our analysis of wild-type cells agrees with previous reports of MreB and FtsZ localization in *R. sphaeroides* (60, 61, 71, 72) that differs from that of *E. coli* (67) and *C. crescentus* (68, 69). By tracking MreB, FtsZ, and PG synthesis (Fig. 4 and 6), we present a model for cell elongation and division in *R. sphaeroides* (Fig. 7B) in which both MreB and PG synthesis are localized to the midcell during early stages of cell elongation, while FtsZ is localized at the cell pole. As wild-type cells elongate, FtsZ becomes colocalized with MreB and components of the elongasome at the midcell. In the late stages of cell division, the septum forms and initiates the process of cell envelope invagination. During this process, MreB moves from the “old” midcell to those in each forming daughter cell, while PG synthesis and FtsZ remain localized at the preexisting midcell. We also propose that, following separation of the daughter cells, MreB redirects PG synthesis and cell elongation to the new midcells, while FtsZ is retained at the new poles of each daughter cell until it depolymerizes and repositions for a new round of division.

This model predicts there are several differences in cell elongation and division between *R. sphaeroides* and other bacteria. In other Gram-negative bacteria, FtsZ and MreB interactions are required for septal PG synthesis or at least are colocalized at the midcell (67, 70, 84). In *E. coli* and *C. crescentus*, FtsZ filaments are proposed to recruit MreB from the lateral cell wall to the midcell, likely promoting function of PBP2, PBP1A, and/or other proteins required for pre-septal PG synthesis (67). Our data suggest a similar synergistic relationship among MreB, FtsZ, and pre-septal PG synthesis/remodeling in *R. sphaeroides* (Fig. 6B, D and E). Furthermore, the movement of *R. sphaeroides* MreB to the midcell of new daughter cells during the late stages of cell septation supports a conclusion that MreB plays an important early, but not late, role in directing midcell PG biosynthesis that is then directed by FtsZ. In *C. crescentus* and other α-proteobacteria that contain homologs of MreB, midcell localization of this filament during septation appears to be conserved (63, 66, 68, 84). However, the dynamic movement of *R. sphaeroides* FtsZ from the midcell to the new pole and the continued presence of MreB at the midcell suggests MreB may facilitate migration of FtsZ to the midcell at the time of cell division. Furthermore, our data support the interconnected function of MreB and FtsZ and suggest that while cell elongation and division are often considered distinct processes, these modes of PG synthesis are synergistic and contiguous in *R. sphaeroides*.

### The impacts of increased CenKR activity on cell elongation and division

In cells with increased CenKR activity, we observed filamentous and chained cells along with evidence of asymmetric cell division (Fig. 2; Fig. S2). In other Gram-negative bacteria, filamentation is associated with a delay in initiating cell division (85), while chaining is commonly linked to loss of Tol-Pal function and the inability of cells to complete cell division/cytokinesis (14, 86 - 89). Consistent with this, we found that Pal is enriched at poles and exhibited more homogenous localization along the length of cells in strains with increased CenKR activity (Fig. 3). We speculate this is due to a decrease in Pal mobilization pre- and post-division, a property normally attributed to *E. coli* TolQ, TolR, TolA, or TolB loss of function mutants (8, 90). The increase in *tolQRAB* transcript levels in cells with increased CenKR activity (25) leads us to propose that increasing TolQRA-B levels prevent TolB from normally interacting with Pal and diffusing through the cells (Fig. 7C), as predicted by the mobilization-capture model of Tol-Pal function (8). When individual components of the Tol-Pal system were overexpressed in *E. coli*, similar filamentation was only observed when *tolQ* was overexpressed, a phenotype that was proposed to be due to FtsN sequestration by TolQ (91). By increasing the activity of CenKR, we observe impacts predicted to reflect how an increase in TolQRA-B complexes would alter the function of the Tol-Pal system. Moreover, CenKR-dependent control of TolQRA levels may represent a mode of post-translational regulation of Pal’s “primary” (division) and “secondary” (PG remodeling, OM-PG connection, etc.) activities.

We propose that the failure of cells overexpressing *cenK* to properly localize Pal at the division septum delays OM comigration with the IM during midcell constriction thereby delaying/blocking cell division and disrupting periplasmic size/homeostasis (Fig. 7C and D). In cells with increased CenKR activity, we also observed instances where the IM of daughter cells remain connected through narrow channels, suggesting that OM constriction is vital to finishing cytokinesis. Our data also show that cells with increased CenKR activity continue to elongate, initiate cell division, and assemble PG along the length of the cells, likely due to the altered dynamics of MreB and FtsZ localization in this strain. Indeed, previous work has shown that blocking cell division in *R. sphaeroides* by treatment with the FtsI inhibitor cephalexin leads MreB to polymerize throughout the length of the cell, similar to what we observe in cells with increased CenKR activity (60). Thus, our data predict that cells overexpressing *cenK* assemble PG along the length of the cells (Fig. 4) despite having not finished the separation of daughter cells in the prior cell cycle thereby preventing/delaying depolymerization of FtsZ and accumulation of MreB filaments along the length of the bacterium (Fig. 6; Fig. S5; Fig. 7D). This prediction is consistent with our previous finding that the CenKR TCS affects transcription of PG biosynthetic genes clusters, activating genes encoding elongasome components and PG remodeling/hydrolysis enzymes while repressing genes encoding known subunits of the divisome (25).

Given the observed changes in the localization of the Pal lipoprotein and division septum in cells with increased CenKR activity, we propose that overexpression of *cenK* delays cell division by sequestering Pal-PG interactions away from the midcell. Lipoprotein-PG interconnection is known to be vital for the normal interactions between the OM and PG layers as well as regulating periplasmic width (1). However, the *E. coli* Lpp protein, which provides this function in this and many other Gram-negative bacteria, has no known homolog in α-proteobacteria. Disruption of Lpp and OM-PG interconnectivity has been linked to changes in periplasmic distance (92), but in α-proteobacteria periplasmic width has been proposed to result from the linkage of β-barrel proteins to PG in *Rhizobiales* (93). While additional work is needed to understand the interactions of PG synthesis during cell elongation and division in *R. sphaeroides* and other α-proteobacteria, our data suggest that the impacts of CenKR activity on Pal localization play an important role in OM division and integrity, OM-PG stability, as well as secondary roles in PG synthesis, cytokinesis, and ultimately periplasmic homeostasis in *R. sphaeroides*. It is possible that CenKR activity is regulated by the interaction of CenK with an envelope-derived ligand to ensure or respond to Pal-PG and/or OM-PG interactions during cell elongation and division. Alternatively, CenK activity may be repressed/modulated by a periplasmic protein that binds the sensory domain of a HK in response to a given stimulus, thereby acting as a rely on OM-PG or periplasm homeostasis to the IM bound HK (Fig. 7A). This mode of regulation has been documented for other cell envelope stress response TCSs in α-proteobacteria (94 - 97).

In other bacteria, genetic or antibiotic perturbations that inhibit MreB-FtsZ interaction blocks cell division, causes filamentation, and results in regularly spaced FtsZ-rings along the length of the cell (67, 98). These phenotypes are comparable to what we observed in cells overexpressing *cenK* in which Z-rings appear to be “locked” along the length of the cell (Fig. 6). In *E. coli* and *C. crescentus*, these regularly spaced FtsZ-rings can result in improper recruitment of PBPs and MreB-directed TPases such as PBP2 that are required for early septation (67, 68). In contrast, when we treat cells overexpressing *cenK* with A22 that blocks MreB polymerization, cells continue to elongate suggesting that FtsZ accumulation is sufficient to allow cell elongation in the absence of MreB filaments. Furthermore, treatment of cells overexpressing *cenK* with antibiotics that block PBP2 activity (amdinocillin) results in localized swelling suggesting that altered localization of MreB alone is not sufficient to affect PBP2 activity or its movement during elongation. Taken together, this suggests that completion of cell division, specifically, OM constriction and cytokinesis in *R. sphaeroides* is required for FtsZ ring depolymerization and elongasome movement. Additionally, our data suggest that the proposed “locked” Z-rings configuration is sufficient to continue PG synthesis and cell elongation in a PBP2 independent manner suggesting redundant functions of FtsZ coordinated PBPs in *R. sphaeroides* (Fig. 7B). Indeed, previous work has highlighted similar MreB-independent elongation in *C. crescentus* (84, 99) and *E. coli* (100) as directed by FtsZ and its constituents.

This work, together with previous studies of CenKR, has provided important new insights, posed additional questions, and provided approaches that can be used to dissect cell elongation and division in *R. sphaeroides* and other α-proteobacteria. Given the phenotypes observed in cells overexpressing *cenK* in conjunction with the number of genes encoding IM, periplasmic, or OM proteins that are direct or indirect targets of CenKR activity, we propose that the analysis of other regulon members can provide new insights into the dynamic localization of PG biosynthesis, MreB/FtsZ positioning and depolymerization, secondary functions of Tol-Pal, independent movement/function of PBP2, and other division- associated processes. For example, α-proteobacteria like *R. sphaeroides* encode CpoB (which is co-transcribed with *pal*) but lack genes encoding homologs of the key OM lipoproteins LpoA/LpoB. Thus, the important auxiliary role of the TolQRA subcomplex in the regulation of CpoB-LpoB connection and PBP1b’s TPase activity at division septum (6) might not be applicable to *R. sphaeroides* and other α-proteobacteria. In addition, genes encoding a number of lipoproteins of unknown function are differentially expressed in CenKR mutants (25), including one (*RSP_1200*) which when disrupted results in protrusions and lipid secretions from the midcell septum suggesting a previously unrecognized role of this protein in cell division (45). Finally, disruptions in Tol-Pal interactions in other bacteria have been reported to lead to vesicle secretion (89, 101) similar to what we observed either in cells overexpressing *cenK* or in high lipid mutants that contain mutations blocking CenKR function (45). Thus, additional analysis of how CenKR activity impacts these cellular processes can provide important new insights into the lifestyle and evolutionary diversity of *R. sphaeroides*, α-proteobacteria, and broadly Gram-negative bacteria.

## SUPPLEMENTAL MATERIAL

Fig. S1 mbio.00631-23-s0001.tif Cryo-EM imaging of the R. sphaeroides cell envelope. (A and B) Additional measurements of the cell envelope of parent (green box plots) and cenK overexpression cells (purple box plots). Box plots for each strain represent the median (black lines) and interquartile range (boxes) of measurements for the distance between the (A) IM and PG (nm) or (B) OM and PG (nm). (C) Quantification of the division septum of parent and cenK overexpression cells imaged by cryo-EM. For cells with multiple division sites (*n* = 85), instances where the OM-IM appear to remain connected through division (Fig. S1D) or have become separated (Fig. 2F) were tallied, and the percentage of total division sites observed was calculated. (D) (Left panel) A 4,800x magnified 2D cryo-EM image of cells overexpressing cenK. (Middle panel) A central slice from a 3D tomogram of the same cell at 19,500× magnification. For tomogram slices, 10 z-slices were summed, representing a ~9.2 nm thick slice of a binned-by-2 tomogram. A chain of extracellular lipids is seen budding from the cell septum and appears to originate from the OM. (Right panel) A 3D model of the same cell depicting the OM (blue), IM (green), and extracellular vesicles (orange). (E) (Left) A 4,800× magnified 2D cryo-EM image of cells overexpressing cenK with multiple early division septa. (Right) A 19,500× magnification 2D cryo-EM image of another cell showing multiple pre-septal division sites. (F) Another example of cell division irregularities showing extended periplasmic regions on the peripheries of the division site.

Fig. S2 mbio.00631-23-s0002.tif Asymmetric division in cells with increased CenKR activity. (A and C) Density heat maps (left) representing cells with increased (A, +IPTG) and basal cenK expression (C, -IPTG). Heat map points, colored according to density (right axis), correspond to constriction sites (right, white arrows) and were mapped onto a projected cell map representing the average cell perimeters centered at the midcell (left, white outline). (B) Graph representing the longitudinal localization of constriction sites relative to the cell poles (0) and midcell (0.5) of cells with basal (white, -IPTG) and increased cenK expression (red, +IPTG).

Fig. S3 mbio.00631-23-s0003.tif OM and ICM vesicles in cells with increased CenKR activity. (A) 3D cryo-ET reconstruction of a cell with increased CenKR activity. A central slice through a NAD filtered tomographic reconstruction. (B) Inset image from (A, red box) showing the IM, OM, EPS, and OM vesicles. For these tomogram slices, 10 z-slices were summed representing a ~9.2 nm thick slice of a binned-by-2 tomogram. (C) 3D model of the same cell depicting the OM (green), IM (blue), and extracellular vesicles (orange) and predicted ribosomes (purple) and putative phosphate granule (yellow). (D) A zoomed-in view of the 3D model overlaying the tomogram demonstrating protrusions originating at the OM surface. (E) A second central slice through a tomographic reconstruction of the same cell (as in A) highlighting ICM morphology at the cell pole and along the length of the cell (green arrows). (F) An additional example of OM budding in cells overexpressing cenK. EPS remains associated with these vesicles.

Fig. S4 mbio.00631-23-s0004.tif Pal localization in wild-type R. sphaeroides. Pal-mCherry spatial localization during the cell cycle as visualized by fluorescence microscopy. (A) Measurements of cell length from microscopy analysis displayed as bee swarm plots (101). Mean length values (μm) from each of the three biological replicates (grey circles, blue squares, and orange triangles) and mean length for each strain (black bar) are shown. For each biological replicate >500 cells were analyzed. Unpaired *t*-tests were used to compare pooled cell length data from the mean values of each biological replicate (*n* =3) (101). N.S. indicate a *P*-value >0.05 and no significant difference. (B) Demographs (left) of Pal-mCherry displaying normalized fluorescence intensity profiles over the length of wild-type cells (y-axis). Fluorescence intensity profile for each cell was combined and sorted by length to model growth and division throughout the cell cycle (x-axis). Cells were also arranged from top to bottom to display the cell pole with the most fluorescence at the top (y-axis). (Right) Representative micrographs with a dotted line showing outlines of the shape of cells, scale bar = 2 μm. Accumulation of Pal-mCherry at the division plane is indicated by an arrow. (C) Violin plots of total cellular fluorescence. Box plots representing population median fluorescence (black line) and biological replicate median values (dots) as well as the interquartile range. * indicates *P* value >0.01, N.S. indicate a *P* value >0.05 and no significant difference as determined by unpaired t-test. (D) Demograph of cells ectopically expressing pal-mCherry. Raw fluorescence profiles for each cell are shown. Cells were sorted by length to model growth and division throughout the cell cycle (x-axis). Cells were arranged from top to bottom to display the cell pole with the most fluorescence at the top relative to the midcell (y-axis).

Fig. S5 mbio.00631-23-s0005.tif Quantification of mCherry-MreB and FtsZ-mCherry foci in pIND5-cenK strains. A. Histogram of calculated mCherry-MreB foci per μm cell length in cells with basal (-IPTG, grey) and increased (+IPTG, gold) cenK expression. Bins = 0.05 μm. Dotted line represents the population mean (-IPTG = 0.65 ± 0.35 μm, *n* = 671; +IPTG = 1.05 ± 0.37 μm, *n* = 1,739, *P* value <0.01). B. Representative bright field (left panels) and fluorescence images (right panels) of pIND5-cenK mreB::mCherry-mreB strains with basal (-IPTG) and increased (+IPTG) cenK expression. C. Histogram of calculated FtsZ-mCherry foci per μm cell length in cells with basal (-IPTG, grey) and increased (+IPTG, gold) cenK expression. Bins = 0.05 μm. Dotted lines represent the population mean (-IPTG = 0.60 ± 0.29 μm, *n* = 1,610; 0.80 ± 0.15 μm, *n* = 79, *P* value >0.05). D. Representative bright field (left panels) and fluorescence images (right panels) of pIND5-cenK ftsZ::ftsZ-mCherry strains with basal (-IPTG) and increased (+IPTG) cenK.

TABLE S1 mbio.00631-23-s0006.docx Primers, plasmids, and strains.

Video S1 mbio.00631-23-s0007.avi Movie of the series of fluorescent images from RADA pulse chase timelapse from wild-type R. sphaeroides cells highlighting midcell synthesis of nascent PG during the cell cycle. Each frame represents a 45-min interval, scale bar = 5 μm.

Video S2 mbio.00631-23-s0008.avi Movie of the series of fluorescent images from RADA pulse chase timelapse from cells overexpressing cenK, highlighting diffuse PG synthesis. Each frame represents a 45-min interval, scale bar = 5 μm.

ASM does not own the copyrights to Supplemental Material that may be linked to, or accessed through, an article. The authors have granted ASM a non-exclusive, world-wide license to publish the Supplemental Material files. Please contact the corresponding author directly for reuse.
