# Supplementary figures and images for: The role of CenKR in the coordination of Rhodobacter sphaeroides cell elongation and division
Source: mBio. 2023 Jun 7;14(4):e00631-23. doi: 10.1128/mbio.00631-23 (PMC10470753; doi:10.1128/mbio.00631-23)

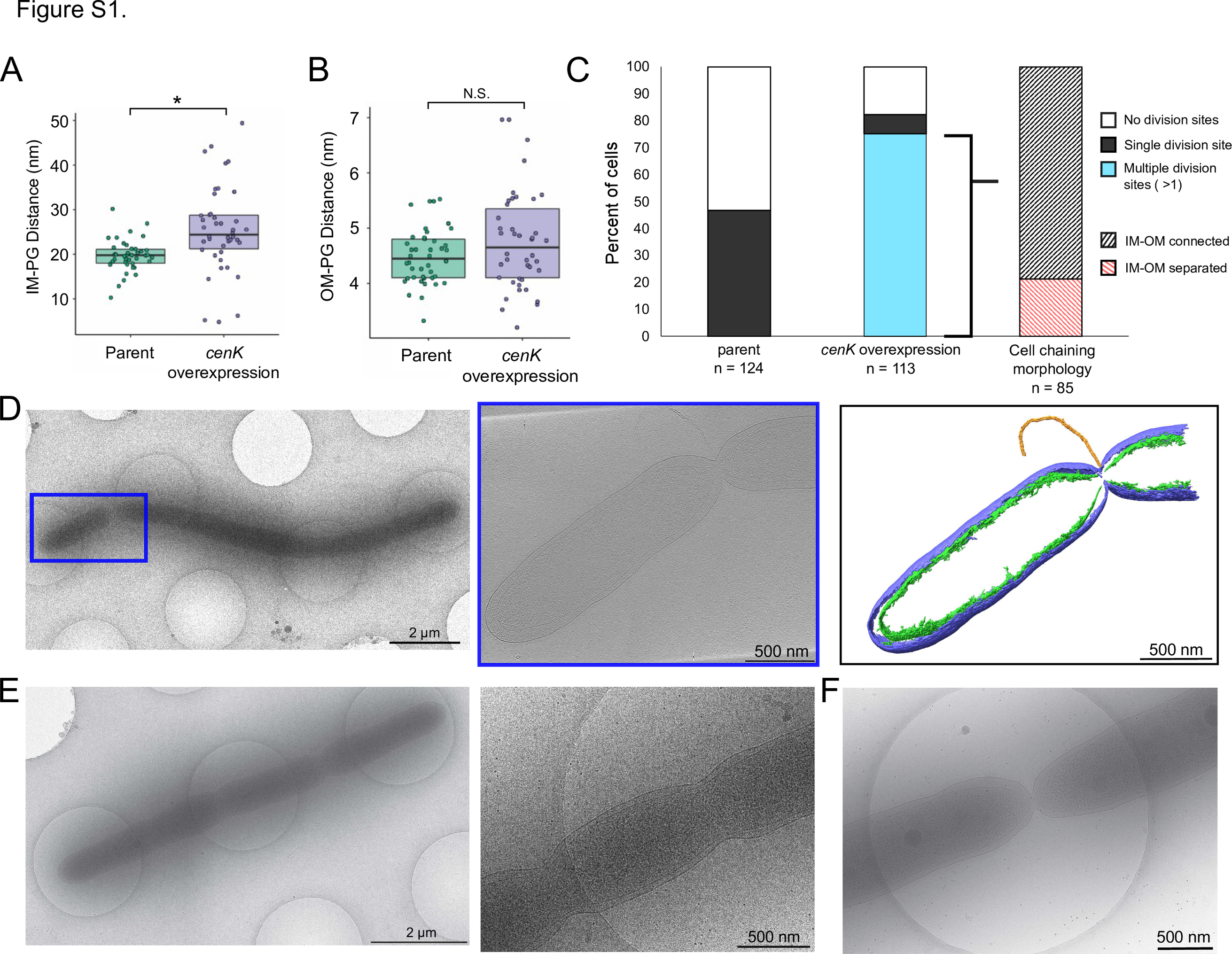

Supplement: Fig. S1 — Cryo-EM imaging of the R. sphaeroides cell envelope. (A and B) Additional measurements of the cell envelope of parent (green box plots) and cenK overexpression cells (purple box plots). Box plots for each strain represent the median (black lines) and interquartile range (boxes) of measurements for the distance between the (A) IM and PG (nm) or (B) OM and PG (nm). (C) Quantification of the division septum of parent and cenK overexpression cells imaged by cryo-EM. For cells with multiple division sites (n = 85), instances where the OM-IM appear to remain connected through division (Fig. S1D) or have become separated (Fig. 2F) were tallied, and the percentage of total division sites observed was calculated. (D) (Left panel) A 4,800x magnified 2D cryo-EM image of cells overexpressing cenK. (Middle panel) A central slice from a 3D tomogram of the same cell at 19,500× magnification. For tomogram slices, 10 z-slices were summed, representing a ~9.2 nm thick slice of a binned-by-2 tomogram. A chain of extracellular lipids is seen budding from the cell septum and appears to originate from the OM. (Right panel) A 3D model of the same cell depicting the OM (blue), IM (green), and extracellular vesicles (orange). (E) (Left) A 4,800× magnified 2D cryo-EM image of cells overexpressing cenK with multiple early division septa. (Right) A 19,500× magnification 2D cryo-EM image of another cell showing multiple pre-septal division sites. (F) Another example of cell division irregularities showing extended periplasmic regions on the peripheries of the division site. [file mbio.00631-23-s0001.tif]

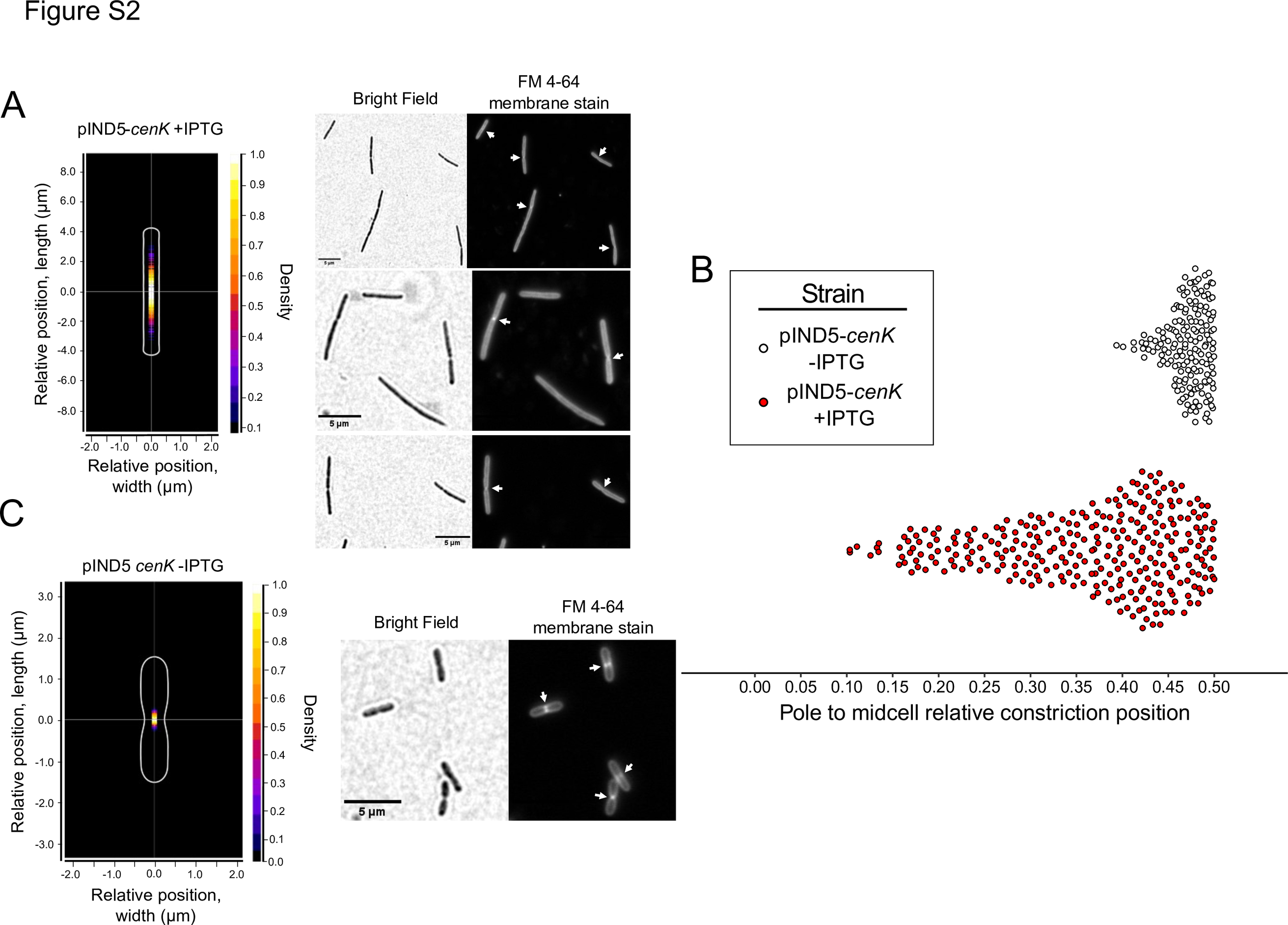

Supplement: Fig. S2 — Asymmetric division in cells with increased CenKR activity. (A and C) Density heat maps (left) representing cells with increased (A, +IPTG) and basal cenK expression (C, -IPTG). Heat map points, colored according to density (right axis), correspond to constriction sites (right, white arrows) and were mapped onto a projected cell map representing the average cell perimeters centered at the midcell (left, white outline). (B) Graph representing the longitudinal localization of constriction sites relative to the cell poles (0) and midcell (0.5) of cells with basal (white, -IPTG) and increased cenK expression (red, +IPTG). [file mbio.00631-23-s0002.tif]

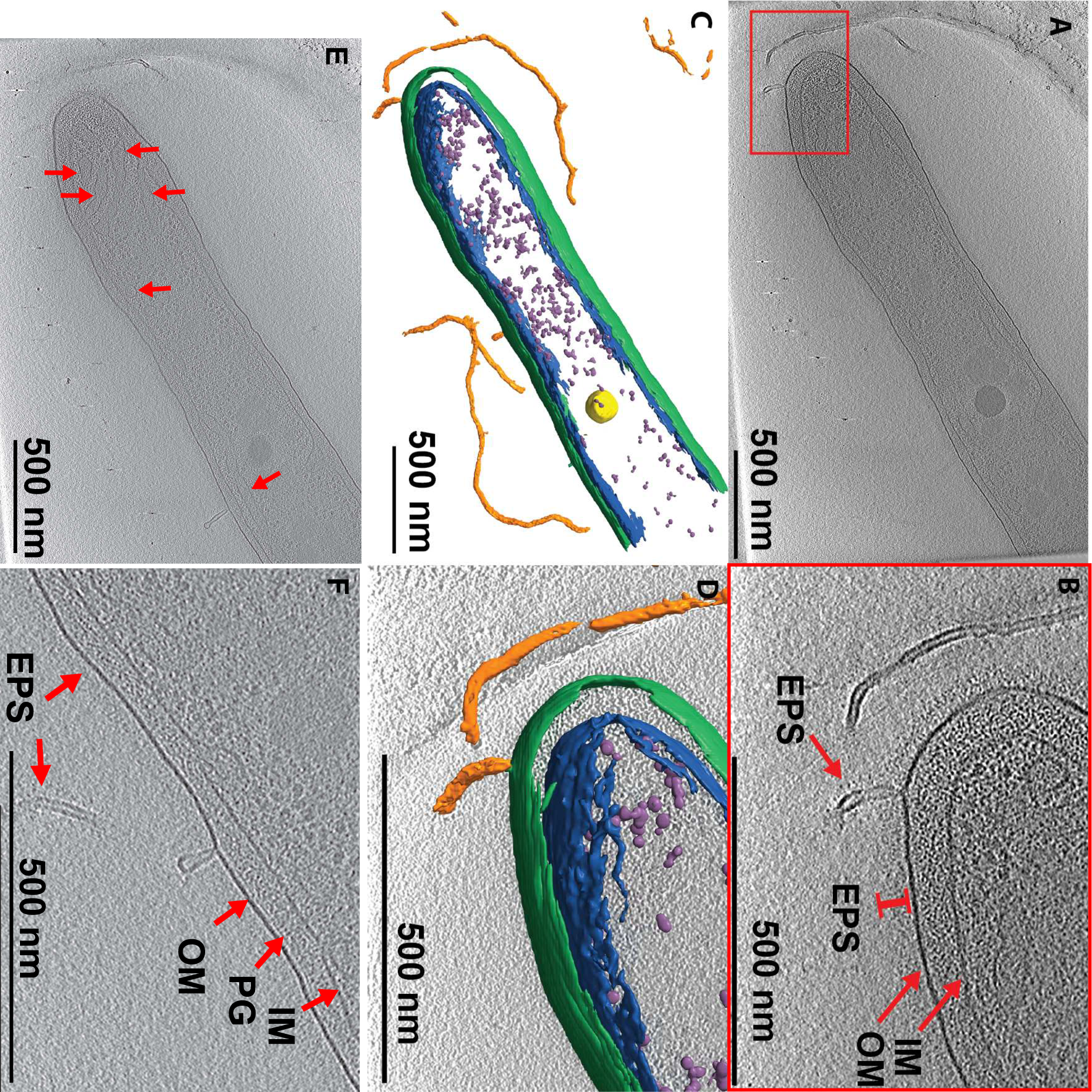

Supplement: Fig. S3 — OM and ICM vesicles in cells with increased CenKR activity. (A) 3D cryo-ET reconstruction of a cell with increased CenKR activity. A central slice through a NAD filtered tomographic reconstruction. (B) Inset image from (A, red box) showing the IM, OM, EPS, and OM vesicles. For these tomogram slices, 10 z-slices were summed representing a ~9.2 nm thick slice of a binned-by-2 tomogram. (C) 3D model of the same cell depicting the OM (green), IM (blue), and extracellular vesicles (orange) and predicted ribosomes (purple) and putative phosphate granule (yellow). (D) A zoomed-in view of the 3D model overlaying the tomogram demonstrating protrusions originating at the OM surface. (E) A second central slice through a tomographic reconstruction of the same cell (as in A) highlighting ICM morphology at the cell pole and along the length of the cell (green arrows). (F) An additional example of OM budding in cells overexpressing cenK. EPS remains associated with these vesicles. [file mbio.00631-23-s0003.tif]

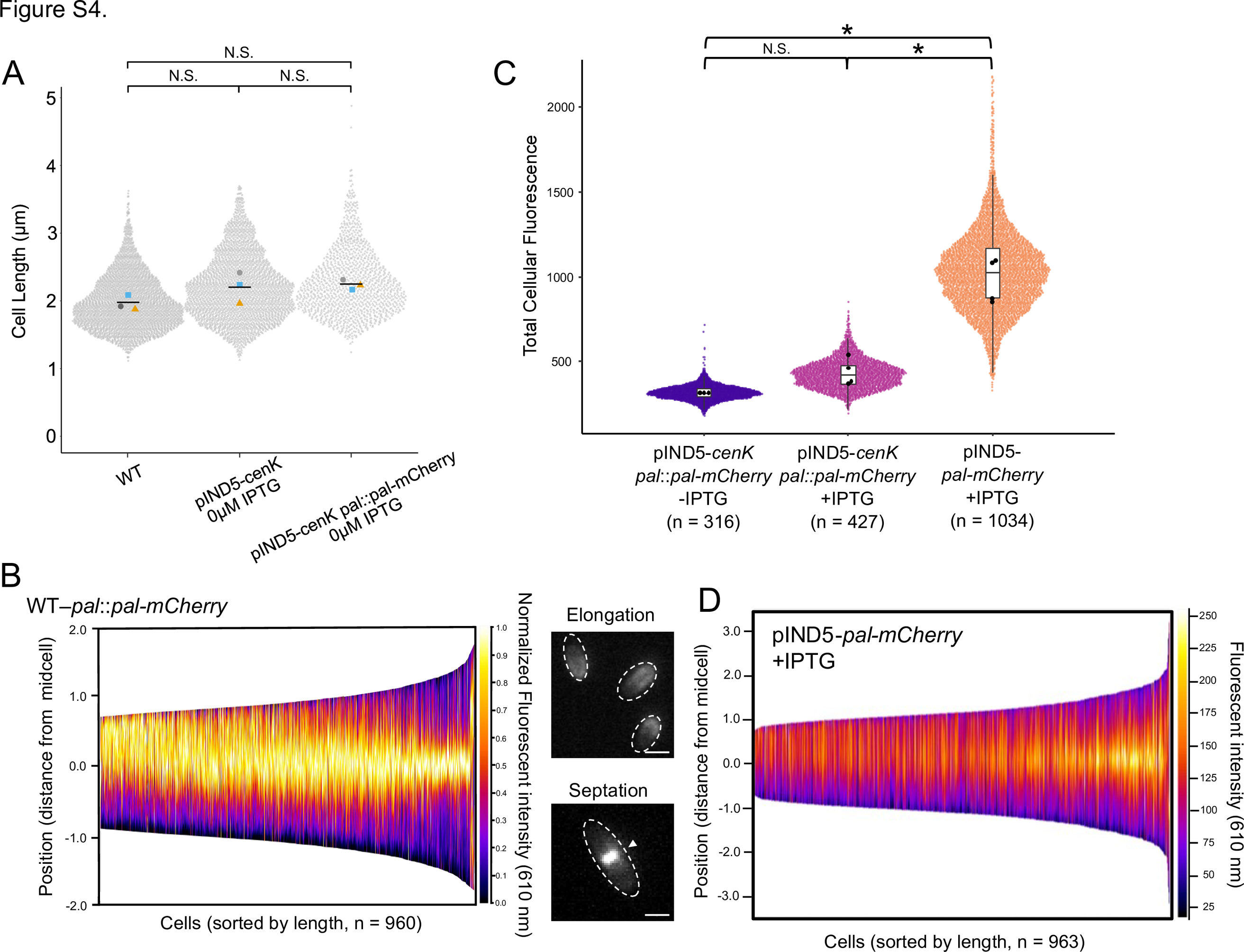

Supplement: Fig. S4 — Pal localization in wild-type R. sphaeroides. Pal-mCherry spatial localization during the cell cycle as visualized by fluorescence microscopy. (A) Measurements of cell length from microscopy analysis displayed as bee swarm plots (101). Mean length values (μm) from each of the three biological replicates (grey circles, blue squares, and orange triangles) and mean length for each strain (black bar) are shown. For each biological replicate >500 cells were analyzed. Unpaired t-tests were used to compare pooled cell length data from the mean values of each biological replicate (n =3) (101). N.S. indicate a P-value >0.05 and no significant difference. (B) Demographs (left) of Pal-mCherry displaying normalized fluorescence intensity profiles over the length of wild-type cells (y-axis). Fluorescence intensity profile for each cell was combined and sorted by length to model growth and division throughout the cell cycle (x-axis). Cells were also arranged from top to bottom to display the cell pole with the most fluorescence at the top (y-axis). (Right) Representative micrographs with a dotted line showing outlines of the shape of cells, scale bar = 2 μm. Accumulation of Pal-mCherry at the division plane is indicated by an arrow. (C) Violin plots of total cellular fluorescence. Box plots representing population median fluorescence (black line) and biological replicate median values (dots) as well as the interquartile range. * indicates P value >0.01, N.S. indicate a P value >0.05 and no significant difference as determined by unpaired t-test. (D) Demograph of cells ectopically expressing pal-mCherry. Raw fluorescence profiles for each cell are shown. Cells were sorted by length to model growth and division throughout the cell cycle (x-axis). Cells were arranged from top to bottom to display the cell pole with the most fluorescence at the top relative to the midcell (y-axis). [file mbio.00631-23-s0004.tif]

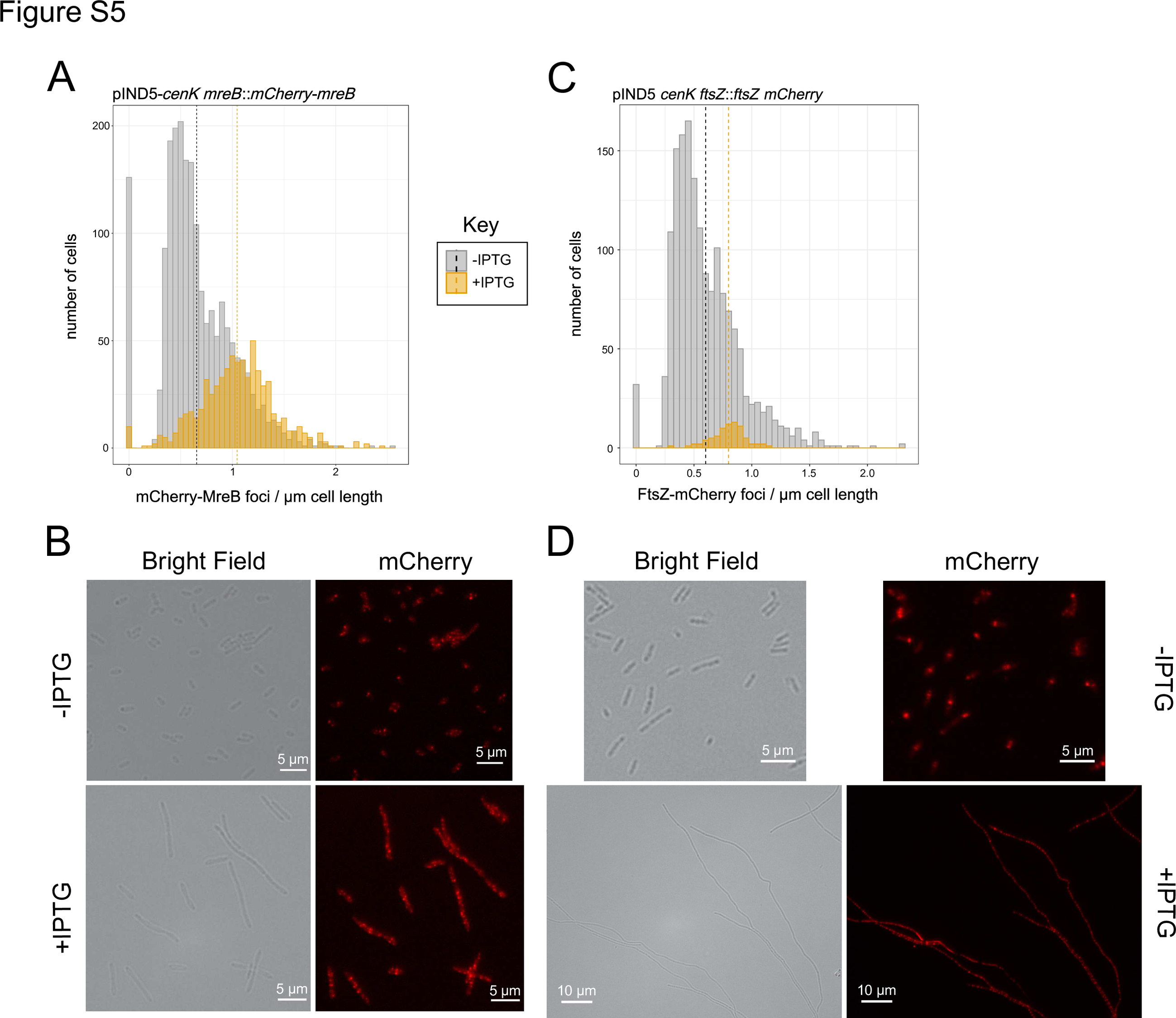

Supplement: Fig. S5 — Quantification of mCherry-MreB and FtsZ-mCherry foci in pIND5-cenK strains. A. Histogram of calculated mCherry-MreB foci per μm cell length in cells with basal (-IPTG, grey) and increased (+IPTG, gold) cenK expression. Bins = 0.05 μm. Dotted line represents the population mean (-IPTG = 0.65 ± 0.35 μm, n = 671; +IPTG = 1.05 ± 0.37 μm, n = 1,739, P value <0.01). B. Representative bright field (left panels) and fluorescence images (right panels) of pIND5-cenK mreB::mCherry-mreB strains with basal (-IPTG) and increased (+IPTG) cenK expression. C. Histogram of calculated FtsZ-mCherry foci per μm cell length in cells with basal (-IPTG, grey) and increased (+IPTG, gold) cenK expression. Bins = 0.05 μm. Dotted lines represent the population mean (-IPTG = 0.60 ± 0.29 μm, n = 1,610; 0.80 ± 0.15 μm, n = 79, P value >0.05). D. Representative bright field (left panels) and fluorescence images (right panels) of pIND5-cenK ftsZ::ftsZ-mCherry strains with basal (-IPTG) and increased (+IPTG) cenK. [file mbio.00631-23-s0005.tif]
