# Supplementary material for: The role of CenKR in the coordination of Rhodobacter sphaeroides cell elongation and division
Source: mBio. 2023 Jun 7;14(4):e00631-23. doi: 10.1128/mbio.00631-23 (PMC10470753; doi:10.1128/mbio.00631-23)
Supplement: TABLE S1 — Primers, plasmids, and strains. [file mbio.00631-23-s0006.docx]

**Table S1 Primers, plasmids, and strains**

1. **Primers**

| **Name** | **Sequence** | **Purpose** |
| --- | --- | --- |
| BDL29-1 | GGAGGATCCAGATCTCATCAC | PCR linearization of pIND5 |
| BDL30-1 | CATATGTAATTTCTCCTCTTTAATTCTAG |  |
| BDL75-3 | AAGAGGAGAAATTACATATGGCTTCCCT  GAAGAAGATCC | PCR amplification of *cenR* (*RSP_0847*) for assembly into pIND5 |
| BDL76-3 | GATGAGATCTGGATCCTCCTCACGCAACA  AGCCTGTAGCCG |  |
| BDL31-1 | AAGAGGAGAAATTACATATGTTGCGGCGGT  TCCTGAAC | PCR amplification of *cenK* (*RSP_1056*) for assembly into pIND5 |
| BDL32-1 | TGAGATCTGGATCCTCCTCAGACGAGGGCGC  TGAG |  |
| BDL33-1 | CTTGTGAGCGGATAACAATGATAC | pIND5 cloning site sequencing and amplification |
| BDL34-1 | CAACCGAGCGTTCTGAACAAATCC |  |
| BDL3-3 | TAGAAAGCCAGTCCGCAGAAAC | PCR linearization of pk18mobsacB |
| BDL3-4 | CTGTCGTGCCAGCTGCATTAATGAATCG |  |
| BDL9-7 | TTAATGCAGCTGGCACGACAGATGGCACTCAA  CCTCATGATG | PCR upstream flank for pk18 *ftsZ*-*mCherry* with GSRS linker |
| BDL50-7 | ATAGATCTGGATCCGTTTGCCTGACGCCGCAGGAAC |  |
| BDL49-7 | AACGGATCCAGATCTATGGTGAGCAAGGGCGAGG | PCR amplification of *mCherry* for pk18 *ftsZ*-*mCherry* with GSRS linker |
| BDL10-7 | GATGTGAACGGATCATTACTTGTACAGCTCGTC |  |
| BDL53-7 | GCTGCCAGCCAGCATTAC | PCR of the 3’ *ftsZ* locus |
| BDL55-7 | GTCCTTTCCGGCTTGTGGTC |  |
| FA204 | tacgaattcCGCTCGGGCGGC | PCR upstream flank for pk18 *pal*-mCherry |
| FA205 | cttgcatgcGCAGGGGTGGGC |  |
| FA206 | tgtacaagtaatgaTCCCATGCGCACCCT | PCR amplification of *mCherry* for pk18 *pal*-*mCherry* |
| FA207 | CTTGCTCACGACCCCCGCACCCAT |  |
| FA208 | GCGGGGGTCGTGAGCAAGGGCGAGGAGGATAA  CATGG | PCR downstream flank for pk18 *pal*-*mCherry* |
| FA209 | CGCATGGGAtcattacttgtacagctcgtcCATGCC |  |
| BDL66-7 | TGCCGTGACATGACATG | PCR of *pal* locus |
| BDL67-7 | GATCTCGGCCTTGATGTCG |  |
| FA334 | gaaattacatatgatgACCCATCTTCCGAAGGC | PCR of *pal* locus for mCherry fusion |
| FA335 | ctaattaagcttatcattacttgtacagctcgtcCATGC |  |
| FA332 | gtacaagtaatgataagcttaattagctgagcttggactcctg | PCR linearization of pIND5 containing *mCherry* |
| FA333 | GAAGATGGGTcatcatatgtaatttctcctctttaattctagatgtgt |  |

| FA169 | tcgagctcggtacccggTCGGCACGGCGATCC | PCR upstream flank for pk18 *mCherry*-*mreB* |
| --- | --- | --- |
| FA170 | aacgacggccagtgccCGCCCGAGATCCGGCCGAC |  |
| FA172 | ggagggggcTCGTTTCTCACCGGCCTCTTCT | PCR amplification of *mCherry* for pk18 *mCherry*-*mreB* |
| FA173 | TGCTCACCATGCTCTGCTGATCCTGTCCTGCGA |  |
| FA174 | TCAGCAGAGCATGGTGAGCAAGGGCGAGG | PCR downstream flank for pk18 *mCherry*-*mreB* |
| FA175 | TGAGAAACGAgccccctccgctgcc |  |
| BDL81-7 | GCGCACCCTTCCTATAAGC | PCR of *mreB* locus |
| BDL82-7 | GCCAGATCAGGAAGATCCC |  |

1. **Plasmids**

| **Name** | **Description** | **Source** |
| --- | --- | --- |
| pk18mobsacB | Broad host range mobilizable vector; Km^R^ oriT(RP4) mobT *sacB lacZα* | (1) |
| pIND5 | Replicable plasmid used for IPTG-inducible expression; Km^R^. | (2) |
| pIND5-*cenR* | pIND5 containing *cenR* (*RSP_0847*) | This work |
| pIND5-*cenK* | pIND5 containing *cenK* (*RSP_1056*) | This work |
| pIND5-*pal*-*mCherry* | pIND5 containing *pal*-*mCherry* translational fusion | This work |
| pk18mobsacB-*pal*-mCherry | pk18 containing the genomic regions directly flanking the 3’ end of *pal* for the insertion of mCherry before the stop codon | This work |
| pk18mobsacB-*ftsZ*-mCherry | pk18 containing the genomic regions directly flanking the 3’ end of *ftsZ* for the insertion of mCherry before the stop codon | This work |
| pk18mobsacB-mCherry-*mreB* | pk18 containing the genomic regions directly flanking the 5’ end of *mreB* for the insertion of mCherry before the start codon | This work |

1. **Strains**

| **Strains** | **Relevant characteristics** | **Source** |
| --- | --- | --- |
| ***Rhodobacter sphaeroides* strains** | | |
| 2.4.1 | Wild type | ATCC-17023 |
| BDL008 | pIND5-*cenR* in 2.4.1 background | This work |
| BDL003 | pIND5-*cenK* in 2.4.1 background | This work |
| BDL072 | *cenR*(D56A) | (3) |
| BDL098 | pIND5-*cenK* *cenR*(D56A) | This work |
| TD000322 | Δ*RSP_0382* (Δ*phaC*) | (4) |
| BDL007 | pIND5-*cenK* Δ*RSP_0382* | This work |
| BDL134 | *pal*::*pal-mCherry* | This work |
| FA285 | pIND5-*pal*-­­­*mCherry* in 2.4.1 background | This work |
| BDL139 | pIND5-*cenK* *pal*::*pal-mCherry* | This work |
| BDL144 | *mreB*::*mCherry*-*mreB* | This work |
| BDL142 | pIND5-*cenK* *mreB*::*mCherry*-*mreB* | This work |
| BDL141 | *ftsZ*::*ftsZ-mCherry* | This work |
| BDL140 | pIND5-*cenK* *ftsZ*::*ftsZ*-*mCherry* | This work |
| ***Escherichia coli* strains** | | |
| DH5α | F- Φ80*lac*ZΔM15 Δ(*lacZYA*-*argF*) U169 *recA*1  *endA*1 *hsdR*17 (rK–, mK+) *phoA* *supE*44 λ– *thi-*1 *gyrA*96 *relA*1 | Bethesda Research Laboratories |
| S17-1 | *TpR SmR recA1 thiE1* *pro-82* *hsdR17* *RP4-2-Tc::Mu-Km::Tn7* λpir | (5) |
| NEB 5-alpha competent *E. coli* | *fhu*A2 Δ(*argF*-*lacZ*)U169 *phoA* *gln*V44 Φ80  Δ(*lacZ*)M15 *gyrA*96 *recA*1 *relA*1 *endA*1 thi-1 *hsdR*17 | New England Biolabs |

**References.**

1. Schäfer A, Tauch A, Jäger W, Kalinowski J, Thierbach G, Pühler A. 1994. Small mobilizable multi-purpose cloning vectors derived from the Escherichia coli plasmids pK18 and pK19: selection of defined deletions in the chromosome of Corynebacterium glutamicum. Gene 145:69–73.

2. Ind AC, Porter SL, Brown MT, Byles ED, de Beyer JA, Godfrey SA, Armitage JP. 2009. Inducible-Expression Plasmid for Rhodobacter sphaeroides and Paracoccus denitrificans. Appl Environ Microbiol 75:6613–6615.

3. Lakey BD, Myers KS, Alberge F, Mettert EL, Kiley PJ, Noguera DR, Donohue TJ. 2022. The essential Rhodobacter sphaeroides CenKR two-component system regulates cell division and envelope biosynthesis. PLOS Genetics 18:e1010270.

4. Yilmaz LS, Kontur WS, Sanders AP, Sohmen U, Donohue TJ, Noguera DR. 2010. Electron Partitioning During Light- and Nutrient-Powered Hydrogen Production by Rhodobacter sphaeroides. Bioenerg Res 3:55–66.

5. Simon R, Priefer U, Pühler A. 1983. A Broad Host Range Mobilization System for In Vivo Genetic Engineering: Transposon Mutagenesis in Gram Negative Bacteria. 9. Nat Biotechnol 1:784–791.
